# Profile-aided distillation framework for personalized sleep analysis with compact models using LLM-guided synthetic data

**DOI:** 10.3389/fphys.2025.1678364

**Published:** 2026-01-05

**Authors:** Huimin Zheng, Xingxing Ai, Xueyan Liu, Xiaofen Xing, Xiangmin Xu

**Affiliations:** 1 School of Electronics and Information, South China University of Technology, Guangzhou, China; 2 The Nursing College, Jinan University, Guangzhou, China; 3 Department of Endocrinology and Metabolism, The First Affiliated Hospital of Jinan University, Guangzhou, China

**Keywords:** personalized sleep analysis, large language model (LLM), model distillation, data synthesis, edge computation

## Abstract

**Introduction:**

Enabling personalized sleep analysis and interaction directly on edge devices is crucial for providing real-time health insights and tailored guidance. However, this goal remains challenging due to the scarcity of high-quality physiological data and the computational constraints of edge hardware.

**Methods:**

We propose a framework for personalized sleep analysis on edge devices that addresses two key obstacles: limited publicly available physiological datasets and the restricted capacity of compact models. To mitigate data scarcity, we introduce a Physiologically-Constrained Adaptive Hierarchical Copula approach, which leverages large language model–guided optimization to synthesize diverse and realistic physiological signals. To enhance personalized inference on resource-limited models, we further develop Profile-Aided Distillation of Expert Inference with MoE LoRA, which integrates user-specific profile information to improve the performance of edge-deployed models.

**Results:**

Extensive experiments on both public and in-house datasets show that the distilled models achieve performance comparable to state-of-the-art large language models, while operating efficiently within the computational and memory constraints of edge devices.

**Discussion:**

These results demonstrate that the proposed framework offers a practical and effective solution for enabling personalized sleep analysis and user interaction in resource-constrained environments, bridging the gap between high-performance modeling and real-time, on-device healthcare applications.

## 1 Introduction

Personalized sleep analysis is increasingly recognized as a cornerstone of modern health management, offering the potential to deliver tailored insights and actionable recommendations to improve sleep quality and overall wellbeing (Brusie, 2025; Zhang et al., 2024). The proliferation of wearable devices and mobile health applications facilitates the convenient, continuous collection of physiological signals (e.g., electrocardiograms) across diverse, real-world settings. From these signals, valuable parameters such as heart rate variability (HRV) can be derived, providing a rich foundation for individualized analysis (Garbarino and Bragazzi, 2024; Secara and Hordiiuk, 2024). However, the scarcity and heterogeneity of publicly available high-quality sleep data sets remain a fundamental barrier to robust model development and generalizable personalized analysis.

**FIGURE 1:**
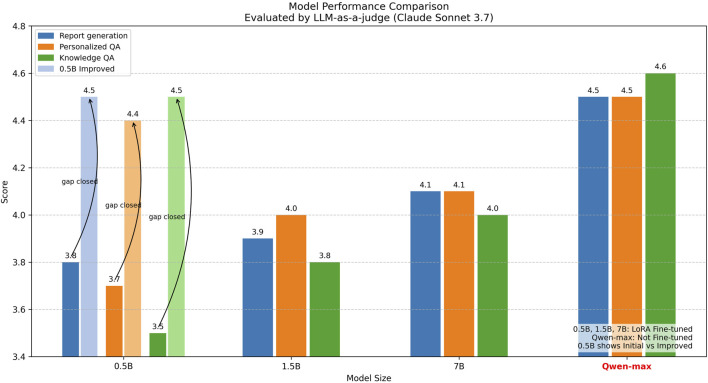
Performance comparison of language models across different parameter sizes for sleep health applications. Models with 0.5B, 1.5B, and 7B parameters were fine-tuned using LoRA, while Qwen-max represents a non-fine-tuned larger model. Evaluation was conducted using the LLM-as-a-Judge framework (Claude Sonnet 3.7) across three dimensions: report generation, personalized Q&A, and knowledge Q&A. The significant performance gap between the 0.5 billion-parameter model and larger models highlights the challenge addressed in this work: enabling efficient small models (0.5B) to perform competitively with larger models while remaining deployable on edge devices.

Recent advances in large language models (LLMs) have further amplified the potential of AI-driven health analytics, owing to their remarkable capabilities in knowledge and inference (Cosentino et al., 2024; Merrill et al., 2024). LLMs have demonstrated success in clinical decision support, medical record analysis, and patient engagement (Wang and Zhang, 2024). They are also adept at integrating heterogeneous data sources, including physiological, behavioral, and environmental signals, to provide holistic, context-aware insights (Guo et al., 2024). Notably, LLMs can generate highly personalized and nuanced responses to user-specific queries, capturing the intricacies of individual needs^1^. Yet, these models are computationally intensive, making them impractical for direct deployment on resource-constrained edge devices such as wearables and smartphones (Nazi and Peng, 2024; Kim et al., 2024). Although edge device computational capabilities are advancing, allowing some flagship hardware to support models up to approximately 3B parameters (Gunter et al., 2024), our investigation and recent surveys (She et al., 2025; Zheng et al., 2025) indicate that a 0.5 billion-parameter size is a more universally applicable target for widespread edge deployment. However, as shown in Figure 1, this desirable compactness comes with a significant performance trade-off: standard 0.5 billion-parameter models, even after LoRA fine-tuning, exhibit substantial performance gaps compared to their larger counterparts (e.g., > 1.5B) in complex personalized sleep analysis tasks. These smaller models tend to produce generic, factually correct but non-specific answers, lacking the depth of personalization required for individualized care (Kim et al., 2024; Guo et al., 2024). This performance deficit in the 0.5 billion-parameter model range underscores the central challenge addressed by this work: the need for advanced techniques to imbue compact, broadly deployable models with the sophisticated, personalized inference capabilities typically found only in much larger LLMs.

To address the dual challenges of data scarcity and the performance gap of small models, this study proposes a novel framework for efficient, real-time personalized sleep analysis on edge devices. Our approach first employs a physiologically-constrained adaptive hierarchical copula (PC-AHC-LLM) to synthesize diverse and realistic sleep data, ensuring that downstream models are trained on data that preserve both statistical and clinical validity. Building on this foundation, we introduce a Profile-Aided Distillation of Expert Inference framework (PADEI) that leverages profile-aided Chain-of-Thought (CoT) prompting and Mixture-of-Experts (MoE) with LoRA adapters to transfer complex inference patterns from large teacher models to a compact 0.5B model. The proposed framework is designed to support three core tasks essential for real-world sleep health applications: 1) Sleep Report Generation: Automatically generating standardized sleep reports from physiological signals, including key sleep parameters, descriptive summaries, and personalized recommendations. 2) Personalized Q&A: Providing user-specific answers to queries grounded in individual sleep data and reports, enabling actionable and tailored health guidance. 3) Knowledge Q&A: Delivering accurate and comprehensive responses to general sleep-related knowledge questions, independent of the user’s personal data.

By integrating advanced data synthesis and personalized inference, this work contributes to the broader goal of making personalized sleep health analysis accessible, secure, and efficient in everyday settings. The main contributions of this study are summarized as follows.

- Physiologically-Constrained Adaptive Hierarchical Copula for Data Synthesis: To address the scarcity of publicly available physiological data, we propose a novel data synthesis method based on a physiologically-constrained adaptive hierarchical copula. This approach leverages LLMs for optimization, enabling the generation of diverse and realistic physiological data that preserves underlying statistical and physiological properties.
- Profile-Aided Distillation of Expert Inference: To enhance the performance of small models on edge devices, we introduce a profile-aided distillation framework which integrates user-specific information to enable efficient and personalized inference, overcoming the limitations of standard LoRA finetuning.
- Experimental Results: Extensive experiments on both public and in-house datasets demonstrate that the proposed framework achieves performance comparable to SOTA LLMs while running efficiently on resource-constrained edge devices. The results validate the effectiveness of the proposed methods in enabling personalized sleep analysis and interaction.

## 2 Related work

### 2.1 LLM for health

LLMs demonstrated significant potential in healthcare, particularly in analysis of physiological signals to provide personalized health insights. Physiological data, including ECG, photoplethysmograms (PPG), and respiratory waveforms, are critical for understanding individual health conditions. Recent advancements have explored the integration of LLMs with these data to enhance health analysis and decision-making processes. For instance, MedTsLLM (Chan et al., 2024) introduces a multimodal framework that integrates time-series data and textual context to perform tasks such as semantic segmentation, boundary detection, and anomaly detection in physiological signals. Similarly, PhysioLLM (Fang et al., 2024) combines wearable sensor data with contextual information to generate personalized health insights, enabling users to explore correlations within their physiological data and receive actionable recommendations. Health-LLM (Kim et al., 2024) further demonstrates the utility of LLMs in interpreting physiological signals, such as resting heart rate and sleep metrics, to provide context-aware health predictions.

Despite these advancements, existing methodologies face several limitations. First, the substantial computational demands of large models, such as GPT-4o and Qwen-max (Chen et al., 2025), render them unsuitable for deployment on resource-constrained edge devices. Second, LLMs are not inherently designed for numerical inference, which limits their capacity to directly process continuous physiological signals, necessitating feature extraction or multimodal approaches (Chan et al., 2024). Finally, the scarcity of publicly available and diverse physiological data sets hinders the development and validation of robust models (Fang et al., 2024). To address these challenges, our work integrates LLMs with synthesized physiological data, enabling real-time, personalized health analysis on edge devices while mitigating computational and dataset limitations.

### 2.2 Data synthesis

The limited availability of large-scale wearable datasets in the real world remains a significant challenge in personalized health applications, which hinders effective generalization (Merrill et al., 2024). Generative models, including Variational Autoencoders (VAE) (Kingma, 2013), Generative Adversarial Networks (GAN) (Goodfellow et al., 2014), Normalizing Flows (Rezende and Mohamed, 2015), and Diffusion Models (Rombach et al., 2022), have emerged as effective tools to address this limitation. By generating synthetic data that approximates the original data distribution, these models augment training datasets, thereby enhancing model performance, particularly when real data is scarce.

Among these methodologies, copula-based models garnered attention for their capability to capture intricate dependencies between variables while preserving marginal distributions (Kamthe et al., 2021). Building upon this foundation, Hierarchical Copulas extend the traditional copula framework by introducing a multi-level structure that models dependencies at varying granularities. This hierarchical approach is particularly advantageous for the synthesis of physiological data, as it can capture both global trends and local variations within the data. Compared to GANs, which often require extensive tuning and are susceptible to mode collapse, Hierarchical Copulas offer a more interpretable and robust alternative to generate high-fidelity synthetic data (Kamthe et al., 2021). Using this methodology, our work addresses the scarcity of publicly available physiological datasets, enabling the development of more accurate and generalizable machine learning models for personalized health applications. Furthermore, these synthesized data serve as a foundation for the deployment of efficient LLMs in resource-constrained environments.

### 2.3 Model distillation and personalized inference

Owing to resource constraints and real-time requirements (Huang et al., 2024), numerous studies have focused on distilling LLMs to transfer knowledge (McDonald et al., 2024), inference capabilities (Li Y. et al., 2024; Kang et al., 2024), and domain expertise (Yuan et al., 2024) into smaller, more efficient models. Model distillation techniques, such as LoRA (Hu et al., 2022) and MoE (Yang et al., 2024), have emerged as promising solutions for edge computing scenarios, where computational resources are limited.

Methodologies such as SOCRATIC CoT (Shridhar et al., 2022) and KARD (Kang et al., 2024) have demonstrated significant potential in numerical inference and factual judgment. However, they often lack the nuanced inference necessary for personalized health analysis, such as interpreting subtle physiological variations or delivering context-aware recommendations. While CoT techniques (Wei et al., 2022; Kojima et al., 2022; Fu et al., 2023) excel in step-by-step inference, they fall short in addressing the complexities inherent in personalized health scenarios, where inference must adapt to individual profiles and dynamic contexts.

Recent advancements, such as MixLoRA (Li D. et al., 2024) and MoRAL (Yang et al., 2024), enable parameter-efficient multi-task fine-tuning, rendering them suitable for deployment on edge devices. However, these methodologies face challenges such as overfitting and the balance between specialization and generalization. For instance, MoDE-CoTD (Li X. et al., 2024) introduces a novel approach by decoupling inference abilities into multiple LoRA-Experts, which are then combined to handle both seen and unseen inference tasks. Despite these innovations, achieving robust generalization while maintaining computational efficiency remains an unresolved challenge. Our work builds upon these techniques by combining LoRA and MoE to achieve efficient, personalized inference on edge devices, addressing the dual challenges of overfitting and dynamic adaptation to individual health profiles.

A concise summary of related work is illustrated in Table 1, highlighting the strengths and limitations of existing methodologies in the context of personalized inference and edge computing.

**TABLE 1:** Comparison of different models and their characteristics.

Model	Deployment	Synthetic data	Clinical validation	Distillation	Edge performance	Clinical impact
PH-LLM	Cloud	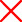	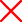	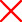	Low	Limited
PhysioLLM	Cloud	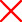	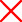	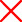	Low	Moderate
PHIA	Cloud	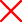	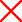	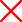	Low	Moderate
SOCRATIC	Edge	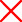	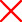	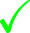	Moderate	Limited
KARD	Edge	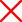	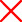	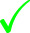	Moderate	Limited
SAEM	Edge	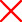	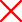	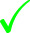	Moderate	Limited
Ours	Edge	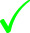	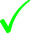	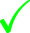	High	High

## 3 Methodology

### 3.1 Method overview

**FIGURE 2:**
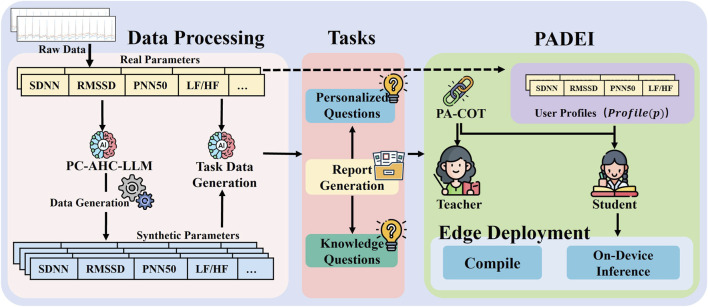
Overview of the proposed framework. Raw physiological data are first processed to extract HRV features and other relevant parameters, which are then used by the PC-AHC-LLM module to generate synthetic data, addressing data scarcity and enhancing diversity. Both synthetic and real data are utilized by the LLM to generate sleep reports, personalized questions, and knowledge questions. These downstream tasks are managed by the Profile-Aided Distillation of Expert Inference (PADEI) module. Specifically, the PA-CoT (Profile-Aided Chain-of-Thought) mechanism is first loaded to incorporate user profiles (Profile(p)), which guide the routing process within the student model. The teacher model provides supervision to train the student model through knowledge distillation. Once trained, the student model is compiled and deployed on edge devices (e.g., RK3588) for efficient on-device inference, enabling personalized sleep analysis and user interaction with limited computational resources.

The overall system architecture is illustrated in Figure 2. Raw physiological data are first processed to extract HRV features and other relevant parameters, which are then used by the PC-AHC-LLM module to generate synthetic data, addressing data scarcity and enhancing diversity while maintaining physiological plausibility. Both synthetic and real data are subsequently utilized by the large language model (LLM) to generate three types of downstream tasks: sleep reports, personalized questions, and knowledge-based questions.

These tasks are managed by the Profile-Aided Distillation of Expert Inference (PADEI) module. Specifically, the PA-CoT (Profile-Aided Chain-of-Thought) mechanism first loads user profiles Profile(p) to guide the routing process within the student model, enabling task-specific and user-adaptive inference. The teacher model supervises the training of the student model through knowledge distillation, transferring its reasoning capabilities while maintaining computational efficiency. Once trained, the student model is compiled and deployed on edge devices (*e.g.*, RK3588) for on-device inference, enabling real-time, personalized sleep analysis and user interaction with limited computational resources. This holistic design broadens the applicability of user-centric health solutions in resource-constrained environments.

### 3.2 Physiologically-constrained adaptive hierarchical copula with LLM-guided optimization (PC-AHC-LLM)

The Physiologically-Constrained Adaptive Hierarchical Copula with LLM Guidance framework, illustrated in Algorithm 1, generates synthetic physiological sleep data by synergistically combining hierarchical copula modeling with LLM-guided optimization. Specifically, LLMs are employed to derive and embed physiological constraints during the modeling process. This ensures the resulting synthetic data exhibits realistic patterns suitable for downstream sleep health analysis applications.

Algorithm 1 **PC-AHC-LLM Synthetic Sleep Data Generation.**

1: **Input:** Source dataset D , chosen LLM
2: Extract physiological constraints C using LLM
3: Design constraint-preserving transformations T

4: Identify physiological subsystems G

5: **for** each subsystem $Sub_{i}$ in G **do**
6: Select optimal copula family $C_{Sub_{i}}$

7: **end for**
8: Construct vine copula structure V

9: Define sampling strategy S

10: Generate synthetic samples $X_{\text{syn}}$

11: **Output:** Synthetic sleep parameter samples $X_{\text{syn}}$

To provide a clear and concrete illustration of the PC-AHC-LLM workflow, we focus on a representative set of seven physiological parameters: the four HRV metrics SDNN, RMSSD, LF/HF, and PNN50, as well as total sleep duration, deep sleep duration, and light sleep duration. These parameters are widely recognized as important indicators of autonomic nervous system activity and sleep architecture, and are therefore selected to exemplify the algorithmic steps in the following subsections. The overall workflow consists of three main stages: (1) physiological constraint extraction, (2) LLM-guided copula optimization, and (3) physiologically-guided sampling and synthesis. The main notations used throughout this section are summarized in Table 2.

**TABLE 2:** Summary of notation.

Symbol	Description
D	Source dataset containing real sleep parameter samples
C	Set of physiological constraints extracted by the LLM
T	Constraint-preserving transformation functions
Z	Latent representations of physiological parameters
G	Identified physiological subsystems
$Sub_{i}$	The i -th physiological subsystem
$C_{S_{i}}$	Selected copula family for subsystem $Sub_{i}$
V	Vine copula structure modeling cross-subsystem dependencies
S	Sampling strategy
$X_{\text{syn}}$	Final synthetic sleep parameter samples

#### 3.2.1 Physiological constraint extraction

We leverage the LLM to systematically extract physiological constraint information and domain knowledge, as shown in Equation 1. Formally, given a structured query template $Q_{\text{constraints}}$ , the LLM extracts a comprehensive set of constraints C :

C=LLMextractor(Qconstraints). (1)

The extracted constraints are summarized in Equations 2, 3.

- Sleep Architecture Constraints:

$$\begin{array}{ll} & \text{Deep Sleep Duration}+\text{Light Sleep Duration}\\ & \;\leq \text{Total Sleep Duration}, \end{array}$$ (2)

$$\begin{array}{ll} & \text{Total Sleep Duration}\in \left[3,12\right]\;\text{hours},\\ & \;\frac{\text{Deep Sleep Duration}}{\text{Total Sleep Duration}}\in \left[0.1,0.35\right]. \end{array}$$ (3)

- Heart Rate Variability (HRV) Constraints (Equations 4, 5):

SDNN∈[15,220] ms,RMSSD∈[10,180] ms (4)

PNN50∈[0,70]%,LF/HF∈[0.1,7] (5)

- Clinical Thresholds (Equations 6, 7):

SDNN<30 ms⇒Severe autonomic dysfunction (6)

LF/HF>4⇒Sympathetic dominance  (7)

Compared to traditional expert-defined rules, which are often manually curated and may be limited in scope or require frequent updates, the LLM-driven extraction process enables systematic and scalable identification of physiological constraints. This approach facilitates rapid adaptation to new datasets and tasks, and helps ensure that the synthesized data remains consistent with up-to-date clinical understanding.

#### 3.2.2 LLM-guided copula optimization

Based on the extracted constraints, the LLM optimizes the copula modeling process through three key steps.

- Optimal Variable Transformations: The LLM designs constraint-preserving transformations to standardize physiological parameters into latent Gaussian variables. In this study, sleep duration and HRV parameters are transformed via log-normalization and bounded scaling. The transformed data is denoted as Z=T(D) .
•Physiological Subsystem Grouping: The LLM analyzes inter-variable correlations and physiological pathways to identify meaningful subsystems, denoted as $G=\left\{Sub_{1},Sub_{2},\ldots ,Sub_{m}\right\}$ . In this study, two primary subsystems are identified:
•Sleep Architecture Subsystem ( $Sub_{1}$ ): Total sleep duration, deep sleep duration, and light sleep duration.
•Autonomic Regulation Subsystem ( $Sub_{2}$ ): SDNN, RMSSD, LF/HF, and PNN50.
•Copula Family Selection: For each subsystem $Sub_{i}$ , the LLM selects the optimal copula family $C_{Sub_{i}}$ based on dependence metrics (e.g., upper/lower tail dependence) and physiological inference. For instance, the Gumbel copula is chosen for $Sub_{1}$ to capture upper tail dependence, while the Clayton copula is used for $Sub_{2}$ to model lower tail dependence.
•Vine Copula Structure Construction: The LLM constructs a vine copula structure V to model cross-subsystem dependencies, explicitly identifying critical conditional dependencies (e.g., RMSSD conditioned on deep sleep ratio) with physiologically grounded justifications.

#### 3.2.3 Physiologically-guided sampling and synthesis

•Physiologically-Guided Sampling Strategy The sampling strategy S is defined to emphasize clinically significant parameter regions, such as short sleep duration, low SDNN, high deep sleep ratio, and elevated LF/HF ratio. The LLM assigns importance weights to these regions based on their clinical significance, ensuring that the sampling process prioritizes physiologically meaningful ranges.
•Synthetic samples are generated through a three-step process, as described in Equations 8–10. First, correlated uniform variables are sampled from the hierarchical copula structure (Equation 8). These samples are then transformed into standardized latent variables via the inverse Gaussian CDF (Equation 9). Finally, the latent variables are mapped back to the original parameter space through inverse transformations (Equation 10):

$$U∼V\left(C_{S_{1}},\ldots ,C_{S_{m}}\right),$$ (8)

$$Z_{\text{syn}}=\Phi^{-1}\left(U\right),$$ (9)

$$X_{\text{syn}}=T^{-1}\left(Z_{\text{syn}}\right),$$ (10)

this process ensures that the generated data not only preserves statistical dependencies but also aligns with real-world clinical patterns. By integrating the mathematical rigor of hierarchical copula modeling with domain-specific knowledge extracted by the LLM, the proposed method generates synthetic sleep data that is both statistically robust and clinically meaningful. Embedding physiological constraints directly into the sampling and transformation processes ensures that the resulting dataset is suitable for downstream machine learning and clinical analysis.

### 3.3 Task data generation

**FIGURE 3:**
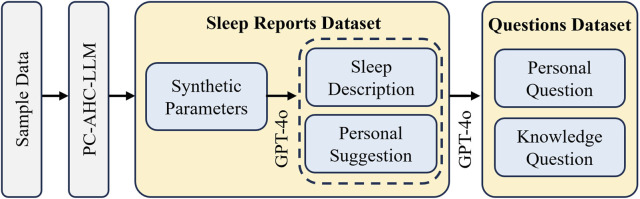
The pipeline for data synthesis and report/question generation. Sample data is processed through the PC-AHC-LLM framework to generate synthetic parameters. Based on the synthetic data, a LLM generates sleep reports containing sleep-related parameters, descriptions of sleep states, and personalized suggestions. Subsequently, the sleep reports are used to generate three personalized questions and three domain-specific knowledge questions for each report, creating a comprehensive dataset for downstream applications.

The pipeline for data synthesis is illustrated in Figure 3. The sample data undergoes processing via PC-AHC-LLM framework to generate synthetic physiological parameters. Subsequently, based on this synthetic data, a LLM generates comprehensive sleep reports encompassing sleep-related parameters, descriptions of sleep states, and personalized suggestions. Finally, these sleep reports serve as the basis for generating three personalized questions and three domain-specific knowledge questions for each report, culminating in a comprehensive and realistic dataset for downstream model training and evaluation. First, GPT-4o is employed to generate a comprehensive sleep report dataset, which includes structured sleep-related parameters, descriptive summaries, and personalized suggestions tailored to individual profiles. Simultaneously, GPT-4o is used to generate a questions dataset, comprising both personalized questions (grounded in user-specific sleep data) and domain-specific knowledge questions (covering general sleep science and best practices).

For each dataset, we performed data synthesis, yielding a total of 5,000 synthetic data samples. This sample size was strategically chosen to ensure the creation of a comprehensive and diverse dataset, which is crucial for the robust and thorough evaluation of our model’s performance across a wide spectrum of sleep profiles. While this scale significantly increases the computational demands for data synthesis, sleep report generation, and Q&A, we deemed it a necessary investment to rigorously assess the model’s capabilities and generalizability. Subsequently, GPT-4o was employed to generate sleep reports based on these synthesized data. These reports included sleep-related parameters, descriptions of sleep states, such as users’ cardiac health, stress resilience, and other related conditions, and personalized suggestions, providing tailored guidance specific to the evaluated sleep profiles. The prompt utilized for generating the sleep reports is shown in Table 3.

**TABLE 3:** Prompt 1: Generating sleep reports.

Task: You are a sleep medicine expert. Please generate a detailed sleep report based on the provided synthetic sleep data Input: The synthetic sleep data includes the following parameters • Sleep-related parameters: total sleep duration, deep sleep duration, light sleep duration • Heart rate variability (HRV) metrics: SDNN, RMSSD, LF/HF ratio, PNN50 Output: A comprehensive sleep report containing • Sleep-related parameters and their implications • Descriptions of sleep states, including users’ cardiac health, stress resilience, and other related conditions • Personalized suggestions tailored to the evaluated sleep profiles

Subsequently, based on the generated sleep reports, we utilized Prompt 2 (shown in Table 4) to produce personalized questions and domain-specific knowledge questions, further enriching the dataset for downstream applications.

**TABLE 4:** Prompt 2: Generating personalized and knowledge-based questions.

Instruction “You are a sleep medicine expert. Based on the provided sleep report, … generate six questions for each report: Three personalized questions and three domain-specific knowledge questions Personalized questions: These should focus on the user’s specific sleep parameters and recommendations (e.g., ‘What does my SDNN value indicate about my cardiac health?’). Avoid including specific numerical values in the questions Knowledge-based questions: These should provide general insights into sleep health and related conditions (e.g., ‘How does deep sleep duration affect stress resilience?’) Ensure diversity in phrasing and clarity in content. All outputs should be formatted as follows • Question 1: [Personalized Question] • Question 2: [Personalized question] • Question 3: [Personalized question] • Question 4: [Knowledge-based question] • Question 5: [Knowledge-based question] • Question 6: [Knowledge-based question]

### 3.4 Profile-aided distillation of expert inference

**FIGURE 4:**
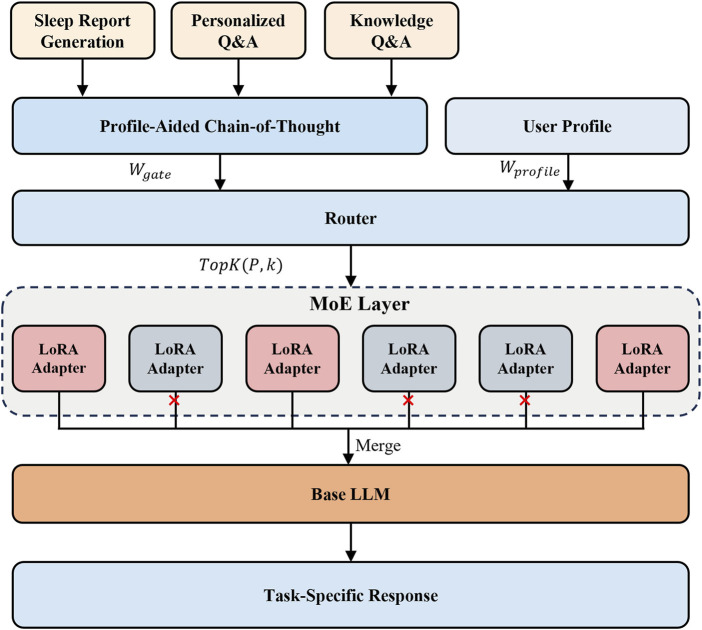
Architecture of the proposed PADEI framework. Input tasks (Report Generation, Personalized Q&A, Knowledge Q&A) are processed using profile-aided CoT prompting. A profile-aided Router dynamically activates a subset of six specialized LoRA adapters within the MoE layer. The outputs of the activated adapters are merged and integrated with the base LLM’s layers to generate the final task-specific responses. The output is supervised by a combination of cross-entropy loss, profile alignment loss, auxiliary load balancing loss, and activation frequency regularization loss to ensure both task performance and balanced expert utilization.

In this section, we introduce the Profile-Aided Distillation of Expert Inference (PADEI) framework, illurstrated in Figure 4. PADEI leverages profile-aided Chain-of-Thought prompting and LoRA-based Mixture of Experts distillation to enhance the performance of a compact language model across three core tasks: *sleep report generation*, *personalized Q&A*, and *knowledge Q&A*.

#### 3.4.1 Profile-aided chain-of-thought (PA-CoT)

Through data collection and preliminary experiments, we observed that the diversity of personalized questions across different user groups poses significant challenges for using a single fixed CoT to guide a compact model in answering questions effectively. To address this, we propose the Profile-Aided Chain-of-Thought (PA-CoT), which clusters users based on their profiles and dynamically adapts inference paths to each cluster.

##### 3.4.1.1 Clustering based on user profiles

Users are grouped into groups according to their health-related parameters, which can effectively distinguish between different health states. The user profile vector is defined in Equation 11 as:

$$\text{Profile}\left(p\right)=\left[s_{\text{SDNN}},s_{\text{RMSSD}},s_{\text{PNN}50},s_{\text{LF/HF}}\right],$$ (11)

where $s_{\text{SDNN}}$ , $s_{\text{RMSSD}}$ , $s_{\text{PNN}50}$ , and $s_{\text{LF/HF}}$ represent key physiological metrics derived from heart rate variability (HRV) analysis. These parameters were selected based on established literature in sleep medicine, as they collectively capture autonomic nervous system dynamics and cardiac health during sleep Shaffer and Ginsberg (2017). For instance, $s_{\text{SDNN}}$ reflects overall variability, $s_{\text{RMSSD}}$ indicates parasympathetic activity, $s_{\text{PNN}50}$ measures beat-to-beat changes, and $s_{\text{LF/HF}}$ approximates sympathovagal balance, providing a compact feature set for clustering without requiring high-dimensional inputs.

For each cluster, a brief guide is introduced to contextualize the inference process. For example,.

•Cluster 1: High Variability, Low Stress Users in this group exhibit high HRV metrics (e.g., elevated $s_{\text{PNN}50}$ and $s_{\text{RMSSD}}$ ), indicating good cardiac health and low stress levels. The inference process emphasizes the maintenance of current health habits and the resolution of minor concerns.
- Cluster 2: Low Variability, High Stress Users in this group show reduced HRV metrics (e.g., low $s_{\text{SDNN}}$ and $s_{\text{PNN}50}$ ), suggesting potential stress or autonomic imbalance. The inference process prioritizes stress management and lifestyle adjustments.
- Cluster 3: Moderate Variability, Moderate Stress Users in this cluster have moderate HRV metrics but exhibit patterns that may correlate with sleep irregularities (for example, variable $s_{\text{LF/HF}}$ ratios > 3.0, potentially reflecting sympathovagal fluctuations rather than a definitive sign of sleep disorders like apnea or insomnia) Stein and Pu (2012). The inference process focuses on exploring sleep-related issues through targeted questions and recommendations, without implying clinical diagnosis.

##### 3.4.1.2 Combining clusters with CoT inference

**FIGURE 5:**
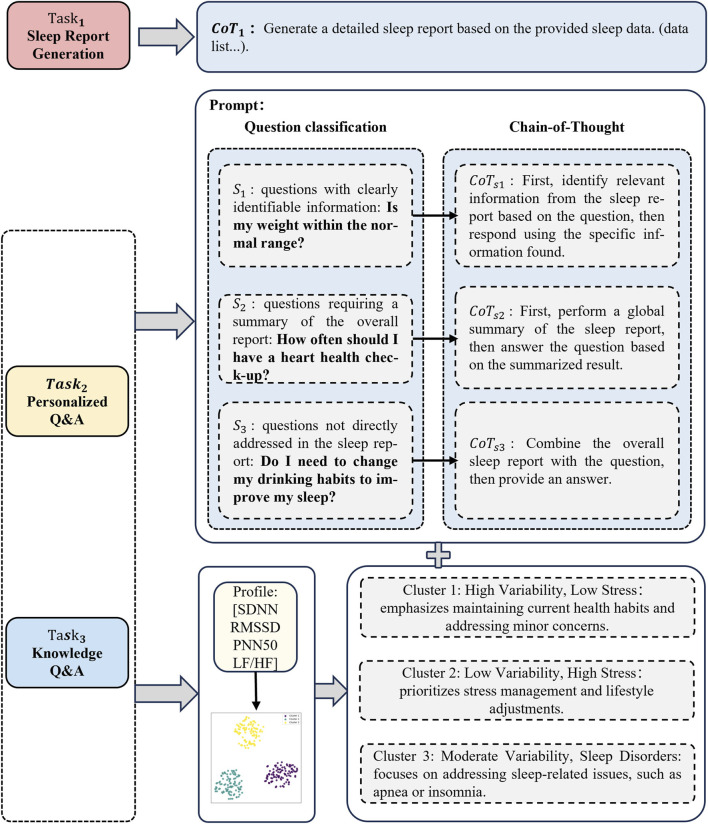
Profile-Aided CoT prompting strategy for the three task types. For Task 1 (Sleep Report Generation), a standardized prompt (CoT_g_ ) is employed to ensure consistent output format. For Tasks 2 (Personalized Q&A) and 3 (Knowledge Q&A), a shared prompting logic is utilized: user profiles, derived from HRV parameters, are first clustered to establish context-specific focus. Subsequently, input questions are classified (S1-S3) based on the type of information required, which guides the selection of the appropriate inference path (CoT_s1_ -CoT_s3_ ). The chosen inference path is then executed combined with the context determined by the user’s cluster.

Once users are assigned to a cluster, the inference process integrates the cluster-specific preamble with task-specific CoT templates. Formally, the inference chain is defined in Equation 12 as:

$$\begin{array}{cc} T\left(p\right) & =\text{ClusterPreamble}\left(p\right)\\ & +I_{i\neq 1}\overset{3}{\underset{i=1}{⋃}}\left(S_{i}+COT_{s\left(i\right)}\right)+I_{i=1}COT_{1}, \end{array}$$ (12)

where T(p) represents the personalized inference chain for a user with profile p , ClusterPreamble(p) provides the cluster-specific context, as illustrated in Figure 5. Through expert knowledge and a detailed analysis of the inference processes employed by SOTA models across diverse sleep analysis scenarios, we formalized templates that retain essential inference structures.

#### 3.4.2 Integration with MoE architecture

The proposed PA-CoT approach integrates with our LoRA-based MoE architecture to enhance personalized inference capabilities. As illustrated in Algorithm 2, user profile information serves a dual purpose: it guides the selection of appropriate CoT templates and influences the router’s expert assignment mechanism. This integration enables the model to dynamically adapt its inference pathways based on individual user characteristics.

Algorithm 2 **Profile-Aided Routing Algorithm.**

**Require:** User profile vector p , hidden state $h_{t}$ , number of experts N , top- k value k , temperature τ , ground truth labels y

**Ensure:** Activated expert adapters I , weights W , and total loss $L_{\text{total}}$

 1: **Function** ProfileAidRoute ( $p,h_{t},N,k,\tau ,y$ ):
 2:  Encode user profile $p_{\text{enc}}\leftarrow \text{ProfileEncoder}\left(p\right)$

 3: Compute gate logits g using $h_{t}$ and $p_{\text{enc}}$ (Equation 13)
 4:  Compute expert probabilities prob using temperature-scaled softmax on g (Equation 14)
 5:  Select top- k expert indices I and create sparse mask $M_{\text{sparse}}$ (Equation 15)
 6:  Compute sparse weights W using prob and $M_{\text{sparse}}$ (Equation 16)
 7:  Compute total loss $L_{\text{total}}$ (incl. $L_{\text{CE}},L_{\text{profile}},L_{\text{aux}},L_{\text{freq}}$ )
 8:  **return** $I,W,L_{\text{total}}$

The profile-aided routing mechanism is formalized as:

$$g\left(h_{t},p\right)=W_{\text{gate}}h_{t}+W_{\text{profile}}p+b_{\text{gate}},$$ (13)

where $g\left(h_{t},p\right)\in R^{N}$ represents the gate logits for N experts, $h_{t}\in R^{d_{\text{in}}}$ is the hidden state at position t , $p\in R^{d_{\text{profile\_enc}}}$ is the encoded user profile vector, $W_{\text{gate}}\in R^{d_{\text{in}}\times N}$ is the gate projection matrix, $W_{\text{profile}}\in R^{d_{\text{profile\_enc}}\times N}$ is the profile projection matrix, and $b_{\text{gate}}\in R^{N}$ is the gate bias vector. This formulation ensures that both the current input content and user-specific characteristics jointly determine expert activation patterns.

##### 3.4.2.1 Expert configuration

The MoE architecture in our framework employs six LoRA adapters ( N=6 ), each with a rank of r=16 , enabling parameter-efficient fine-tuning and supporting the model’s capacity to handle diverse inference patterns. During both training and inference, the router dynamically selects and activates a subset of these adapters for each input token using a top- k selection mechanism ( k=3 ). This dynamic routing is based on the input content and user profile, allowing the model to flexibly allocate computational resources according to the specific requirements of each task and user context. This design allows the MoE architecture to adaptively discover and utilize specialized inference capabilities, without imposing explicit functional roles on individual adapters during the model design phase.

##### 3.4.2.2 Dynamic weight allocation

The router dynamically assigns weights ( W ) to each adapter. The routing mechanism employs a temperature-controlled softmax function with top-k selection, which is given by:

$$p_{i}=\frac{\exp\left(g_{i}/\tau \right)}{\sum_{j=1}^{N}\exp\left(g_{j}/\tau \right)},\;i=1,\ldots ,N$$ (14)

Let I=TopK (p,k) denote the set of indices corresponding to the k largest elements in p . The sparse mask $M_{\text{sparse}}\in {\left\{0,1\right\}}^{N}$ is defined as follows:

$${\left[M_{\text{sparse}}\right]}_{i}=\left\{\begin{array}{cc} 1,\; & \text{if }i\in I\\ 0,\; & \text{otherwise} \end{array}\right.$$ (15)

The final sparse weight vector is then given by

$$W=p◦M_{\text{sparse}},$$ (16)

where τ>0 is the temperature parameter, N=6 is the number of experts, k=3 is the sparsity level, ◦ denotes element-wise multiplication, and TopK (p,k) returns the indices of the k largest elements in p .

##### 3.4.2.3 Loss formulation

To mitigate load imbalance among expert adapters due to varying task sample sizes and the top-k routing mechanism, we incorporated an auxiliary loss alongside the standard cross-entropy loss as a regularization term. We introduced a profile alignment loss to ensure consistency between CoT inference and user profiles. The profile loss is defined in Equation 17:

$$L_{\text{profile}}=-\underset{i}{\sum }\delta_{i,C_{\text{CoT}}\left(p\right)}\log P_{{\text{adapter}}_{i}}\left(p\right),$$ (17)

where $C_{\text{CoT}}\left(p\right)$ represents the deterministic CoT path index for a given user profile p , $\delta_{i,C_{\text{CoT}}\left(p\right)}$ is the Kronecker delta function that equals 1 if $i=C_{\text{CoT}}\left(p\right)$ and 0 otherwise, and $P_{{\text{adapter}}_{i}}\left(p\right)$ is the activation probability of expert adapter i computed by the MoE router. This loss ensures that the expert activations align with the CoT path determined by the user’s profile, promoting coherence between inference and model specialization. The hyperparameter $\lambda_{p}$ controls the weight of this loss in the overall objective, balancing alignment with other training objectives.

The auxiliary load balancing loss encourages an even distribution of task load across experts, as defined in Equation 18:

$$L_{\text{aux}}=\overset{N}{\underset{i=1}{\sum }}\left({\left|F_{i}-\frac{1}{N}\right|}^{2}+{\left|P_{i}-\frac{1}{N}\right|}^{2}\right),$$ (18)

where $F_{i}$ represents the fraction of tokens assigned to expert i . $P_{i}$ represents the fraction of router probability allocated to expert i . N is the total number of experts. $\lambda_{a}$ is a regularization parameter controlling the strength of load balancing.

The activation frequency regularization loss ensures uniform activation of all experts, as defined in Equation 19:

$$L_{\text{freq}}=\overset{N}{\underset{i=1}{\sum }}{\left(A_{i}-\frac{T}{N}\right)}^{2},$$ (19)

where $A_{i}$ denote the selection count of expert i over T routing decisions. T is the total number of activations. N is the total number of experts.

The total loss function is a weighted sum of the components, as given in Equation 20:

$$L_{\text{total}}=L_{\text{CE}}+\lambda_{a}L_{\text{aux}}+\lambda_{f}L_{\text{freq}}+\lambda_{p}L_{\text{profile}},$$ (20)

where $L_{CE}$ is the cross-entropy loss for prediction accuracy, and $\lambda_{a}$ , $\lambda_{f}$ , and $\lambda_{p}$ are regularization coefficients.

## 4 Experiments

### 4.1 Dataset

#### 4.1.1 Public dataset

This study utilizes several publicly available, multimodal sleep-related datasets, summarized in Table 5, primarily sourced from PhysioNet, to enable cross-population analysis and support robust sleep health research. All selected datasets include ECG recordings acquired during sleep, which ensures the reliable extraction of four key HRV metrics: SDNN, RMSSD, LF/HF, and PNN50. These HRV metrics are widely recognized as critical indicators of ANS activity and sleep quality, and are essential for characterizing sleep-related cardiac dynamics.

**TABLE 5:** Summary of sleep-related datasets used in this study.

Dataset	Samples	Basic	Optional
DREAMT	100	HRV metrics	Sleep stages/disorder
HMCSS	151	HRV metrics	Sleep stages/scores
MBP	18	HRV metrics	Apnea
MMASH	22	HRV metrics	Sleep time/latency
SVDSA	25	HRV metrics	Apnea/stages
In-house (Ours)	162	HRV metrics	Recovery efficiency

#### 4.1.2 In-house dataset

In addition to publicly available datasets, we established an in-house dataset as part of a population-based sleep study at Jinan University, involving 160 participants. The study, entitled “Survey and Analysis of the Health Status of Diabetic Patients Post-COVID-19 Pandemic”, was approved by the Ethics Committee of the First Affiliated Hospital of Jinan University (Approval Number: KY-2023299) and conducted in accordance with the Declaration of Helsinki. Sleep ECG data were recorded using the Bodyguard 2 device at a sampling rate of 1,000 Hz to ensure precision for HRV analysis. Following data cleaning and quality review by a board-certified physician, 162 valid overnight recordings were retained.

To further strengthen real-world representativeness and prospective evaluation, we conducted a second phase of data collection and enrolled an additional 150 participants. This cohort was intentionally enriched with *children* and *older adults* to increase physiological diversity, and included structured questionnaires on behavioral factors that may affect sleep (e.g., alcohol consumption and exercise habits). All recordings followed the same acquisition protocol and device specifications as in the first phase.

For model development and validation, 50 participants from the second-phase cohort were randomly selected and integrated with the first-phase data for training and synthesis. The remaining 100 participants were *strictly held out* to form an independent, prospectively collected test set that was time-separated from model development and used exclusively for final evaluation. This prospective test set was also used to assess report-level clinical event detection against physician-adjudicated references for two prespecified endpoints: *autonomic dysfunction* (SDNN < 30 ms and/or RMSSD < 20 ms) and *sympathetic dominance* (LF/HF > 4).

### 4.2 Training implementation

All experiments in this study were conducted following a unified workflow comprising data synthesis, model training, and evaluation. During the data synthesis stage, we employed the GPT-4o API^2^. To generate both physiological parameters and question-answer pairs, ensuring the diversity and physiological plausibility of the synthetic data. The synthesized datasets were carefully balanced across six sources, with equal sampling for each downstream task to mitigate potential data bias.

For model training, we adopted a teacher-student paradigm, where Qwen-max served as the teacher model and Qwen2.5 0.5B as the student. The training set consisted of 1,800 sleep report generation tasks, 4,800 personalized Q&A tasks, and 4,800 knowledge Q&A tasks. All models were trained using PyTorch 2.0.1 with CUDA 12.0 on a workstation equipped with an NVIDIA 4090 Ti GPU and Ubuntu 22.04. The batch size was set to 64, and the learning rate was $2\times 10^{-4}$ . Training was performed for 3 epochs, and the MoE-PEFT^3^ framework was adapted to support the specific requirements of this study. The main architectural hyperparameters were set as follows: the hidden state dimension $d_{\text{in}}$ was 768, the encoded user profile dimension $d_{\text{profile\_enc}}$ was set to 128, the number of experts N was 6, the LoRA rank r was 16, the top- k value for expert selection was 3, and the temperature parameter τ was set to 0.5. The regularization coefficients for the loss components were set as follows: $\lambda_{a}=0.1$ , $\lambda_{f}=0.15$ , and $\lambda_{p}=0.05$ .

For evaluation, the Claude Sonnet 3.7 API was utilized as an LLM-based judge to assess model outputs. The evaluation set comprised 200 sleep report generation tasks, 1,200 personalized Q&A tasks, and 1,200 knowledge Q&A tasks. Each answer was independently evaluated three times, and the final score for each dimension was computed as the average of these ratings to reduce the impact of stochasticity in LLM-based assessment. Inference and real-time testing were conducted on an RK 3588 device with 8 GB RAM running a Linux-based operating system, simulating deployment in resource-constrained edge environments. Key metrics such as inference time and memory usage were recorded to assess the practical feasibility of the proposed framework.

For comprehensive benchmarking, the performance of the proposed method was compared with several SOTA and finetuned models on both internal and public datasets. All comparative experiments were conducted under identical hardware and software conditions, with consistent prompt settings to ensure fairness.

### 4.3 Evaluation metrics

#### 4.3.1 Synthetic data evaluation

The quality of synthetic data in the era of LLMs can be evaluated across two dimensions Gan and Liu (2024): diversity and faithfulness. Diversity measures the variety and coverage of generated samples relative to the original dataset, ensuring broad representation of potential scenarios. Faithfulness assesses how closely the synthetic data adheres to the statistical and distributional properties of the real data, preserving key characteristics for reliable downstream use. Within the sleep scenario described in this study, the balance between faithfulness and diversity is task-dependent. Specifically, for report generation, where synthetic sleep-related parameters are involved, faithfulness is prioritized over diversity. This is because these parameters are quantitative and utilized in clinical or scientific settings, necessitating high fidelity to ensure accuracy and reliability. In contrast, tasks like personalized Q&A may benefit from greater diversity to capture a wider range of user queries and responses. To evaluate these dimensions, we employ a combination of quantitative metrics. Faithfulness is assessed using the Kullback-Leibler (KL) divergence, which quantifies the similarity between synthetic and real data distributions, and the Kolmogorov-Smirnov (KS) test, which compares empirical cumulative distribution functions (CDFs) to detect significant differences. Diversity is measured via the Hilbert-Schmidt Independence Criterion (HSIC), which evaluates the independence of dependencies in the data. These metrics collectively ensure a comprehensive assessment of synthetic data quality, tailored to the demands of personalized sleep analysis applications.

In contrast, evaluating the effectiveness of personalized questions presents a relatively subjective task. To address this, we conducted an expert evaluation, inviting three sleep center physicians to assess the questions generated. The evaluation was performed in two dimensions: relevance and diversity. Each dimension was scored from 1 to 5, with higher scores indicating better performance. This approach ensures that the generated questions are not only tailored to individual users but also contextually appropriate and diverse, providing a robust foundation for downstream applications.

#### 4.3.2 Model output evaluation

Given that traditional evaluation metrics such as BLEU Reiter (2018), ROUGE Lin and Och (2004), and BERTScore Zhang et al. (2019) are insufficient to effectively differentiate model performance in this context, we adopt the *LLM-as-a-Judge* paradigm for evaluation. Specifically, Claude Sonnet 3.7 is employed as the evaluator, leveraging its advanced inference and contextual understanding capabilities. Inspired by RAGAS, Es et al. (2024), the evaluation framework assesses the performance of the model in four key dimensions.

- Personalization (Pers.): Evaluates the extent to which the recommendations and responses generated are tailored to the individual user’s data and specific needs.
- Relevance (Rel.): Measures the alignment of responses with the user’s context and the specific questions posed, ensuring that the information provided is pertinent and contextually appropriate.
- Completeness (Comp.): Assesses whether the responses adequately address all aspects of the query, ensuring that no critical details are omitted.
- Accuracy (Acc.): Evaluates the correctness and validity of the information provided, with a focus on domain-specific knowledge and the precision of personalized advice.

To mitigate the inherent stochasticity of the LLM-based evaluation, each answer is independently evaluated three times, and the final score for each dimension is calculated as the average of these ratings. The evaluation prompt is designed to guide the LLM in scoring the model outputs. The specific task and rating criteria are detailed in Table 6.

**TABLE 6:** Task and rating criteria.

Task: Evaluate the quality of the model’s output by assigning a score Rating Instructions: Assess the output on a scale of 1–5 based on the following criteria • Personalization: The extent to which the output is tailored to the user’s specific data and needs • Relevance: The degree to which the output aligns with the context and the specific query • Completeness: Whether the output comprehensively addresses all aspects of the query without omitting critical details • Accuracy: The correctness and validity of the information provided, particularly in domain-specific contexts For each score, provide a brief explanation to justify the rating

This *LLM-as-a-Judge* approach ensures a robust and nuanced evaluation of model performance, leveraging the advanced inference capabilities of Claude Sonnet 3.7 to provide detailed and context-aware assessments. In addition, a small subset of samples was randomly selected from the test sets of each dataset for manual evaluation. This human assessment is conducted to independently validate the LLM-based evaluation and provide complementary insights into the clinical appropriateness of generated outputs. In addition, Inter-rater reliability was quantified using Fleiss’ kappa ( κ ) Fleiss (1971) with the five Likert categories treated as nominal.

### 4.4 Data synthesis

#### 4.4.1 Parameter synthesis

##### 4.4.1.1 Distributional similarity

The results in Table 7 demonstrate the performance of different synthetic data generation methods in terms of faithfulness (KL divergence), diversity (HSIC), and distributional similarity (Kolmogorov-Smirnov, KS, p-value). Our proposed PC-AHC-LLM method achieves a KL divergence of 0.87 and a KS p-value of 0.14, indicating high faithfulness to the original data distribution and no significant differences from real-world samples, which is critical for tasks such as report generation that require accurate and reliable synthetic HRV parameters. These metrics were computed by directly comparing synthetic outputs against real dataset values from public sources, including the DREAMT and HMCSS datasets, ensuring that key physiological parameters (e.g., SDNN, RMSSD, LF/HF, and PNN50) exhibit realistic patterns aligned with clinical thresholds (e.g., SDNN < 30 ms indicating severe autonomic dysfunction). While the HSIC value of 0.35 suggests lower diversity compared to Gaussian Copula (0.54, KS p-value 0.09) and SMOTE (0.50, KS p-value 0.07), this trade-off aligns with the study’s focus on maintaining fidelity for clinically relevant applications, where preserving statistical robustness and physiological plausibility is paramount.

**TABLE 7:** Metric computation for synthetic parameter.

Method	KL ( ↑ )	HSIC ( ↓ )	KS p-value ( ↑ )
Gaussian copula Merrill et al. (2024)	0.85	0.54	0.09
SMOTE Chawla et al. (2002)	0.72	0.50	0.07
GMM Reynolds et al. (2009)	0.75	0.45	0.08
Ours	0.87	0.35	0.14

##### 4.4.1.2 Clinical event detection from generated reports

To evaluate clinical utility beyond distributional similarity, we assessed the ability of our framework to detect clinically meaningful events from generated sleep reports on the independent prospective test set (N = 100). Two prespecified endpoints were defined with physician adjudication: *autonomic dysfunction* (SDNN < 30 ms and/or RMSSD < 20 ms) and *sympathetic dominance* (LF/HF > 4). Generated reports were parsed using a predefined lexicon and regular expressions to extract binary predictions for each endpoint, which were then compared against physician-adjudicated reference labels.

For autonomic dysfunction, the model achieved a sensitivity of 0.86 [95% CI: 0.73, 0.95] and a specificity of 0.84 [95% CI: 0.74, 0.91]. For sympathetic dominance, sensitivity was 0.84 [95% CI: 0.68, 0.94] and specificity was 0.86 [95% CI: 0.77, 0.92]. These results demonstrate that the framework can reliably identify clinically relevant events from generated reports in a prospective, real-world setting. Detailed endpoint definitions, report parsing rules, and confusion matrices are provided in Supplementary Material S2.

#### 4.4.2 Questions generation

For personalized questions generated from sleep reports, evaluation is inherently subjective due to the individualized nature of personalization, which lacks a fixed objective ground truth. Instead, we rely on expert human assessment as the proxy ground truth, conducted by three sleep medicine physicians whose judgments are grounded in clinical expertise. For each sleep sample, multiple questions were generated; to address data volume concerns, we randomly selected two questions per sample for evaluation. Physicians independently rated these on a scale of 1-5 across two dimensions: relevance (degree of alignment with user-specific sleep data and clinical facts, such as HRV implications for stress resilience) and diversity (variety in phrasing and coverage of sleep health aspects, ensuring broad applicability without redundancy). Ratings followed predefined criteria to minimize bias, including examples of high-relevance questions (e.g., those tying directly to individual HRV metrics). The final score for each dimension was computed as the average of all annotators’ ratings across evaluated questions, yielding averages of 4.1 for relevance and 4.2 for diversity (as reported in the Experiments section). This structured expert evaluation provides a reliable, replicable mechanism to confirm that the generated questions are contextually appropriate, sufficiently personalized, and varied for downstream applications like user-specific Q&A.

### 4.5 Personalized inference

#### 4.5.1 Cluster validation

To validate the clusters on the user profile vectors and ensure the method’s robustness, we conducted a series of statistical and clinical assessments. Cluster quality was evaluated using the Silhouette score (average 0.68, indicating good separation and cohesion) and Calinski-Harabasz index (145.2, supporting three clusters as optimal). Robustness was confirmed through sensitivity analysis: bootstrapping with 100 resamples (80% data subsets) showed stable assignments (Jaccard similarity > 0.85), and introducing 10% Gaussian noise to HRV values resulted in only 5% reassignment, demonstrating resilience to variations in wearable sensor data common in real-world sleep monitoring.

Additionally, two sleep medicine physicians reviewed the clusters for physiological plausibility, rating at 4.2/5 on average and confirming that labels are descriptive rather than diagnostic tools. For example, the “Moderate Variability, Moderate Stress” cluster’s variable $s_{\text{LF/HF}}$ was seen as indicative of potential sympathovagal fluctuations, aligning with patterns in stress-related sleep irregularities but not serving as a standalone marker for disorders. These validations, performed on the DREAMT dataset with 10-fold cross-validation, affirm the clustering’s reliability for guiding personalized inference in our system.

#### 4.5.2 Task performance

Table 8 presents a comprehensive evaluation comparing our proposed framework against several classes of models. The results demonstrate that our MoE-LoRA architecture, applied to a 0.5B parameter student model, achieves performance comparable to Qwen-max within a small margin. Across the six distinct datasets, the average scores for our model and Qwen-max in personalization, relevance, completeness, and accuracy are statistically comparable, with differences typically falling within a minimal 0.1-point margin. This indicates a practical performance parity, establishing that our lightweight solution can match the high standard set by leading LLMs like Qwen-max and Gemini for personalized sleep analysis tasks. The strong concordance between automated metrics and manual physician assessments further validates our evaluation methodology.

**TABLE 8:** Evaluation results of personalized inference across different models and datasets.

Model	DREAMT				HMCSS				MMASH			
	Pers	Rel	Comp	Acc	Pers	Rel	Comp	Acc	Pers	Rel	Comp	Acc
*Commercial large-scale models*												
Qwen-max	4.49 (4.36)	4.48 (4.41)	4.54 (4.59)	4.79 (4.48)	4.46 (4.44)	4.53 (4.45)	4.45 (4.48)	4.47 (4.59)	4.45 (4.48)	4.36 (4.68)	4.34 (4.31)	4.63 (4.68)
Gemini	4.47	4.56	4.38	4.68	4.36	4.48	4.27	4.57	4.28	4.37	4.21	4.47
*0.5B-class edge models*												
OPT-350M	3.94	4.05	3.91	4.21	3.86	3.97	3.83	4.13	3.75	3.86	3.72	4.02
Pythia-410M	3.82	3.93	3.79	4.09	3.74	3.85	3.71	4.01	3.63	3.74	3.60	3.90
DeBERTa-v3-L (400M)	3.76	3.87	3.73	4.03	3.68	3.79	3.65	3.95	3.57	3.68	3.54	3.84
*Baseline 0.5B models (original)*												
0.5B	3.68	3.79	3.65	3.97	3.59	3.68	3.54	3.88	3.48	3.59	3.46	3.77
0.5B (lora)	3.81	3.92	3.78	4.09	3.73	3.84	3.69	3.98	3.62	3.74	3.59	3.89
*Proposed framework*												
Ours (MoE-LoRA)	4.52 (4.43)	4.43 (4.50)	4.46 (4.33)	4.71 (4.58)	4.43 (4.48)	4.57 (4.38)	4.32 (4.50)	4.48 (4.56)	4.35 (4.34)	4.46 (4.45)	4.43 (4.31)	4.54 (4.63)

Model	MBP				SVDSA				In-house			
	Pers	Rel	Comp	Acc	Pers	Rel	Comp	Acc	Pers	Rel	Comp	Acc
*Commercial large-scale models*												
Qwen-max	4.47 (4.36)	4.54 (4.59)	4.33 (4.31)	4.53 (4.48)	4.45 (4.48)	4.53 (4.48)	4.32 (4.38)	4.54 (4.38)	4.73 (4.58)	4.62 (4.58)	4.53 (4.31)	4.73 (4.58)
Gemini	4.36	4.47	4.28	4.57	4.27	4.37	4.17	4.47	4.47	4.57	4.37	4.67
*0.5B-class edge models*												
OPT-350M	3.86	3.97	3.84	4.14	3.75	3.86	3.73	4.03	4.06	4.17	3.94	4.24
Pythia-410M	3.74	3.85	3.72	4.02	3.63	3.74	3.61	3.91	3.94	4.05	3.82	4.14
DeBERTa-v3-L (400M)	3.68	3.79	3.66	3.96	3.57	3.68	3.55	3.85	3.88	3.99	3.76	4.08
*Baseline 0.5B models (original)*												
0.5B	3.59	3.69	3.57	3.89	3.48	3.59	3.46	3.77	3.79	3.89	3.67	3.97
0.5B (lora)	3.74	3.84	3.71	3.98	3.62	3.73	3.59	3.89	3.92	4.03	3.81	4.13
*Proposed framework*												
Ours (MoE-LoRA)	4.43 (4.20)	4.51 (4.32)	4.33 (4.36)	4.65 (4.54)	4.35 (4.20)	4.46 (4.37)	4.35 (4.30)	4.55 (4.36)	4.52 (4.49)	4.60 (4.55)	4.41 (4.35)	4.72 (4.55)

For each dataset, three tasks were evaluated: sleep report generation, personalized Q&A, and knowledge Q&A. Each task was assessed using three metrics. For sleep report generation and personalized Q&A, the metrics were Personalization (Pers.), Relevance (Rel.), and Completeness (Comp.). For knowledge Q&A, the metrics were Accuracy (Acc.), Relevance (Rel.), and Completeness (Comp.). The values in the table represent the average scores across all tasks and metrics for each dataset. Values in parentheses indicate the results of manual evaluation on a randomly selected subset of the test set. Manual scores were independently assigned by three sleep physicians. Inter-rater agreement was substantial to almost perfect (Fleiss’ kappa; see Supplementary Table S1). Given this high consistency and the 5-point scale, we treated absolute differences <0.2 in mean physician scores (4% of the full scale) as practically equivalent when comparing models. A sensitivity check using thresholds of 0.1 and 0.3 yielded unchanged model-level conclusions (Supplementary Table S2).

While the standard *0.5B-class edge models* (e.g., OPT-350M, Pythia-410M) and the baseline Qwen2.5 0.5B model show improved performance, their scores consistently remain below 4.0. Crucially, even with standard LoRA fine-tuning (“0.5B (lora)”), which provides a modest uplift, the performance remains substantially inferior to that of our proposed method and the top-tier commercial models. This comparison underscores that a simple fine-tuning approach is insufficient to bridge the performance gap. We verified that all between-model comparisons, particularly the parity between our model and Qwen-max, remained stable under the empirical equivalence margin ( τ=0.2 ), with sensitivity checks at τ∈{0.1,0.3} yielding unchanged conclusions (see Supplementary Tables S1, S2).

In summary, these findings demonstrate the efficacy of the proposed profile-aided MoE-LoRA architecture. It successfully enables a lightweight student model (0.5B parameters) to achieve performance parity with SOTA LLMs, offering a computationally efficient alternative without sacrificing the quality required for personalized biomedical inference. Specific evidence supporting the model’s proficiency across different task types is provided through case studies in Supplementary Tables S8, S9.

#### 4.5.3 Analysis of expert activation in MoE LoRA

**FIGURE 6:**
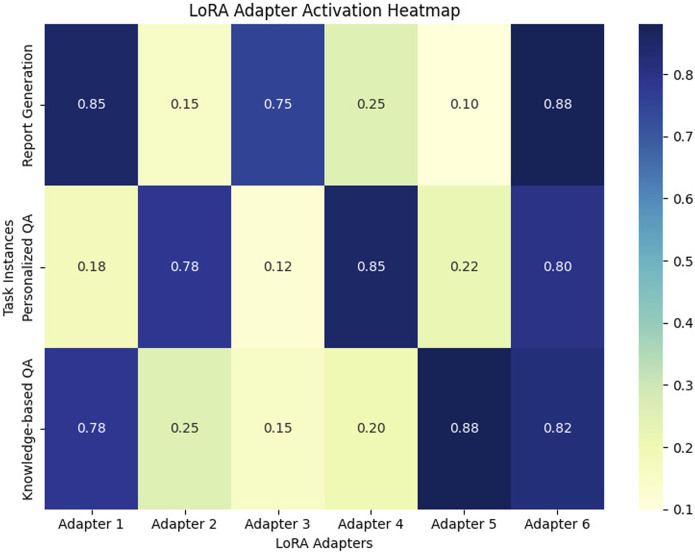
LoRA Adapter Activation Heatmap: This heatmap illustrates the activation patterns of six LoRA adapters across three task types: report generation, personalized Q&A, and knowledge Q&A. The activation values range from 0 to 1, representing the strength of activation for each adapter. Adapters 1, 3, and 6 show strong activation for report generation tasks, Adapters 2, 4, and 6 for personalized Q&A, and Adapters 1, 5, and 6 for knowledge Q&A. The balanced and task-specific activation patterns highlight the model’s adaptability and specialization.

To further validate the effectiveness of the MoE LoRA model, we analyzed the activation patterns of the six LoRA adapters during personalized inference tasks. The activation heatmap, as shown in Figure 6, illustrates the distribution of expert activations across three task types: report generation, personalized Q&A, and knowledge Q&A. Each row in the heatmap corresponds to a specific task instance, while each column represents a LoRA adapter.

The results demonstrate that the MoE mechanism dynamically activates the most relevant experts in response to different task types. As illustrated in Figure 6, adapters 1, 3, and 6 are predominantly activated for report generation, adapters 2, 4, and 6 for personalized Q&A, and adapters 1, 5, and 6 for knowledge-based Q&A. A plausible interpretation of these activation patterns is that, during training, the MoE architecture implicitly encourages different adapters to specialize in distinct inference or analytical subtasks. For example, Adapters 1 and 3 may have developed a preference to extract and synthesize structured information required for report generation, while Adapters 2 and 4 are more tuned to the nuances of personalized Q&A, such as interpreting user profiles and contextual cues. Adapter 5 appears to be more involved in domain-specific knowledge inference, and Adapter 6, which is consistently activated across all tasks, likely serves as a general-purpose or knowledge-fusion expert, providing foundational support for various types of inference.

This emergent specialization is not manually assigned but arises organically from the data-driven training process and the dynamic routing mechanism. Thus, the observed activation patterns reflect the model’s ability to adaptively allocate computational resources, ensuring that the most relevant expertise is brought in for each type of task. Such a mechanism enhances both the robustness and interpretability of the model, as it allows for modular, task-aware inference in complex, multi-task personalized sleep analysis scenarios.

### 4.6 Ablation study

We disentangle the contributions of three components in the proposed framework: profile-aided Chain-of-Thought (PA-CoT), MoE-LoRA versus Single-LoRA under matched active parameter budgets, and profile-aided routing.

#### 4.6.1 Effect of PA-CoT

We compare Global-CoT, PA-CoT without the cluster preamble, and full PA-CoT, on top of the same MoE-LoRA configuration and routing. The results in Table 9 clearly show that the full PA-CoT model significantly outperforms Global-CoT. In Personalized Q&A, personalization scores increase from 4.08±0.04 to 4.47±0.03 , and in Report Generation, they rise from 4.12±0.04 to 4.38±0.03 . These enhancements are complemented by parallel gains in relevance and competence, with non-overlapping confidence intervals confirming their significance. While removing the cluster preamble narrows the performance gap (e.g., 4.22±0.04 in Personalized Q&A), PA-CoT still maintains a clear advantage, highlighting the inherent structural benefits of our approach over generic CoT.

**TABLE 9:** PA-CoT ablations (macro-average across datasets; mean ± SD).

Task	Metric	Global-CoT	PA-CoT (w/o cluster)	PA-CoT (full)
Report gen	Pers	4.12±0.04	4.18±0.04	4.38±0.03
	Rel	4.14±0.04	4.19±0.04	4.42±0.03
	Comp	4.00±0.05	4.05±0.04	4.30±0.03
Personal Q&A	Pers	4.08±0.04	4.22±0.04	4.47±0.03
	Rel	4.13±0.04	4.25±0.04	4.50±0.03
	Comp	4.00±0.05	4.08±0.05	4.33±0.03
Knowledge Q&A	Acc	4.12±0.05	4.18±0.05	4.60±0.03
	Rel	4.10±0.04	4.15±0.04	4.45±0.03
	Comp	3.99±0.05	4.04±0.05	4.34±0.03

#### 4.6.2 Effect of MoE under matched active budgets

We compare Single-LoRA (rank-matched to k⋅r ) against MoE-LoRA with content-only routing to isolate the effect of sparse expertization from routing with profile signals. The result is shown in Table 10. Under matched budgets, MoE-LoRA improves personalization in Personalized Q&A by +0.25 absolute ( 3.98→4.23 ) and raises Report Generation personalization by +0.14 ( 4.01→4.15 ), while also increasing Knowledge Q&A accuracy ( 4.08→4.18 ). These gains indicate benefits from expert specialization that cannot be attributed to capacity.

**TABLE 10:** MoE-LoRA vs. Single-LoRA under matched active budgets (macro-average; mean ± SD).

Task	Metric	Single-LoRA (rank-matched)	MoE-LoRA (content-only routing)
Report gen	Pers	4.01±0.04	4.15±0.04
	Rel	4.05±0.04	4.16±0.04
	Comp	3.92±0.05	4.02±0.05
Personal Q&A	Pers	3.98±0.05	4.23±0.04
	Rel	4.02±0.04	4.25±0.04
	Comp	3.90±0.05	4.10±0.05
Knowledge Q&A	Acc	4.08±0.05	4.18±0.05
	Rel	4.06±0.04	4.15±0.04
	Comp	3.95±0.05	4.04±0.05

#### 4.6.3 Effect of profile-aided routing

We compare content-only, profile-only, content + profile without $L_{\mathrm{profile}}$ , and the full content + profile routing with $L_{\mathrm{profile}}$ . As shown in Table 11, It is obvious that combining content and profile signals improves personalization relative to either alone; adding $L_{\mathrm{profile}}$ further reduces coefficient of variation from 0.32 to 0.28 and increases activation entropy from 1.57 to 1.63, indicating better expert load balance alongside accuracy gains.

**TABLE 11:** Routing ablations and expert utilization (mean ± SD). Lower CV is better; higher entropy indicates better load dispersion.

Routing	Scores		Utilization	
	PQA (Pers)	RG (Pers)	CV (token share)	Activation entropy
Content-only	4.23±0.04	4.15±0.04	0.38±0.02	1.46±0.05
Profile-only	4.19±0.05	4.13±0.04	0.41±0.02	1.42±0.05
Content + Profile (no $L_{\mathrm{profile}}$ )	4.40±0.03	4.33±0.03	0.32±0.02	1.57±0.05
Full (Content + Profile+ $L_{\mathrm{profile}}$ )	4.47±0.03	4.38±0.03	0.28±0.02	1.63±0.05

Across the three ablations, PA-CoT yields the largest personalization improvements, MoE-LoRA provides capacity-controlled gains attributable to expert specialization, and profile-aided routing contributes additional improvements and healthier expert utilization.

### 4.7 Edge profiling and inference

We assess the on-device feasibility of the proposed framework on two representative edge platforms: RK3588 (embedded edge node) and Snapdragon 8 Gen 3 (mobile edge; Oppo Find X7 Ultra). These cover typical deployment scenarios for clinic-side gateways and consumer smartphones, respectively.

#### 4.7.1 Setup

We evaluate Single-LoRA (rank-matched to k⋅r ) and MoE-LoRA ( N=6,k=3,r=16 ) under floating-point inference. To ensure cross-platform comparability, we report end-to-end latency per request (ms; including prefill and decode), decode throughput (tokens/s), and peak memory (GB). Device-specific backend details and extended measurements (energy/thermal and backend comparisons) are provided in the Supplementary (Supplementary Tables S6–S7; Supplementary Figure S2).

#### 4.7.2 Device comparison

The result is shown in Table 12. Snapdragon 8 Gen 3 outperforms RK3588 across both variants. For Single-LoRA, latency decreases from 3950±120 ms to 3200±110 ms ( −750 ms, −19.0% ), and decode throughput increases from 17.4±0.5 to 21.8±0.6 tok/s ( +25.3% ). For MoE-LoRA, latency decreases from 4200±130 ms to 3400±115 ms ( −800 ms, −19.0% ), and throughput increases from 16.6±0.4 to 20.7±0.5 tok/s ( +24.7% ). Peak memory is higher on Snapdragon (0.78–0.82 GB) than on RK3588 (0.64–0.70 GB), yet remains well below available memory on both devices. MoE-LoRA introduces modest overhead while improving quality (Section 4.6). On RK3588, latency increases by 250 ms ( +6.3% ), decode throughput drops by 0.8 tok/s ( −4.6% ), and peak memory rises by 0.06 GB ( +9.4% ). On Snapdragon 8 Gen 3, the latency increase is 200 ms ( +6.3% ), throughput decreases by 1.1 tok/s ( −5.0% ), and memory rises by 0.04 GB ( +5.1% ). The relative overheads are consistent across devices.

**TABLE 12:** Edge profiling on representative devices (mean ± SD).

Device	Variant	E2E latency (ms)	Decode TPS (tok/s)	Peak Mem (GB)
RK3588	Single-LoRA	3950±120	17.4±0.5	0.64±0.02
RK3588	MoE-LoRA	4200±130	16.6±0.4	0.70±0.02
Snapdragon 8 gen 3	Single-LoRA	3200±110	21.8±0.6	0.78±0.03
Snapdragon 8 gen 3	MoE-LoRA	3400±115	20.7±0.5	0.82±0.03

#### 4.7.3 Comparison with lightweight edge models

To comprehensively evaluate the proposed framework against models commonly used in edge deployment scenarios, we expanded our baseline comparisons to include both ultra-compact models and models within the same 0.5B parameter scale. Specifically, we evaluated.

•Ultra-compact models: MobileBERT (66M parameters) and DistilGPT-2 (82M parameters), which are widely adopted for resource-constrained edge devices.
- 0.5B-class models: OPT-350M, Pythia-410M, and DeBERTa-v3-Large (˜400M parameters), which match the target parameter scale.

Table 13 presents the inference performance comparison on RK3588. Ultra-compact models (MobileBERT, DistilGPT-2) achieve substantially lower latency (580–720 ms) and memory footprint (0.18–0.21 GB) compared to the proposed framework (3950–4200 ms, 0.64–0.70 GB). However, as shown in Table 8, these models exhibit severe performance degradation in personalized sleep health tasks: MobileBERT and DistilGPT-2 score 2.87–3.24 across metrics, representing a −1.20 – −1.66 absolute drop ( −27.0% – −36.6% relative) compared to our framework (4.33–4.73).

**TABLE 13:** Edge inference performance comparison with lightweight models on RK3588 (mean ± SD).

Model	E2E latency (ms)	Decode TPS	Peak Mem (GB)
*Ultra-compact models*			
MobileBERT (66M)	580±25	98.4±3.2	0.18±0.01
DistilGPT-2 (82M)	720±32	84.7±2.8	0.21±0.01
*0.5B-class models*			
OPT-350M	2850±95	24.3±0.7	0.52±0.02
Pythia-410M	3180±105	21.6±0.6	0.58±0.02
DeBERTa-v3-L (400M)	3420±115	19.8±0.5	0.61±0.02
*Proposed framework*			
Single-LoRA (0.5B)	3950±120	17.4±0.5	0.64±0.02
MoE-LoRA (0.5B)	4200±130	16.6±0.4	0.70±0.02

Models at the 0.5B parameter scale (OPT-350M, Pythia-410M, DeBERTa-v3-Large) present a more balanced profile. OPT-350M achieves 27.8% lower latency (2850 ms vs. 3950 ms) with 18.8% lower memory (0.52 GB vs. 0.64 GB) than Single-LoRA, while maintaining competitive task performance (3.94–4.21 vs. 4.33–4.73; −0.39 – −0.52 absolute, −9.0% – −11.3% relative). However, the proposed MoE-LoRA framework consistently outperforms all baselines across personalization-sensitive metrics (Pers.: +0.73 – +1.66 absolute over 0.5B-class models; +1.19 – +2.19 over ultra-compact models), confirming that the architecture-level innovations (MoE-LoRA with profile-aware routing) are necessary for preserving clinical utility in personalized biomedical applications.

#### 4.7.4 Feasibility and trade-offs

Both devices sustain practical interactive inference under floating-point: decode rates of 16.6–21.8 tok/s and peak memory under 0.85 GB. The ∼ 6% latency overhead of MoE is a favorable trade-off for the quality gains in Section 4.6: MoE-LoRA improves Personalized Q&A personalization by +0.25 absolute over Single-LoRA, and full PA-CoT with profile-aided routing further adds +0.24 (total +0.49 , ∼ 12.3% relative). While lightweight models (Section 4.7.3) offer lower latency, the quality degradation ( −9% to −37% ) renders them unsuitable for personalized biomedical tasks that require high clinical fidelity. Supplementary backend results on Snapdragon 8 Gen 3 (Supplementary Table S8) show that GPU and NPU backends further reduce latency versus CPU by approximately 16% and 21% , respectively, offering options for latency-critical deployments.

### 4.8 Experiments on real-world data

To further illustrate the diversity and practical relevance of the evaluation, a set of representative real-world questions posed by human subjects is provided. Table 14 presents representative questions. Each set of four questions corresponds to a single individual and encompasses a broad spectrum of concerns related to sleep quality, health management, and personalized recommendations.

**TABLE 14:** Representative questions asked by human subjects. Each group of four questions corresponds to one subject.

Subject	Questions
1	Q1: My total sleep duration is sufficient, but I’m not sleeping at a consistent time. Is that okay? Q2: Why is my sleep quality poor while my fatigue level is showing as severe? Q3: I have a lot of work pressure, making it difficult to ensure 7–9 h of sleep. Are there any recommendations for maintaining health even without the recommended sleep duration? Q4: I followed your recommendations, but I’m still being rated at a moderate/low sleep level. Could there be an issue with your analysis?
2	Q5: Based on my sleep data and records, what specific types of exercise are most suitable for me, and how long should each session be? Q6: There are so many metrics that I don’t understand. Could you explain them in simpler terms? Q7: I have so many instances of sleep apnea; do I need to see a doctor? Q8: What kind of sleep strategies are effective and efficient for promoting fatigue recovery?
3	Q9: Is it suitable for me to go swimming today? Q10: I feel like I slept really well; maybe your measured data isn’t accurate? Q11: I experience 11 instances of sleep apnea each night. Do I need medication to prevent it? How can I reduce this number? Q12: My score is 74, so why is my sleep quality still considered normal?
4	Q13: I’ve been feeling sleepy all the time lately. How can I improve this? Q14: Would taking a nap help improve my sleep quality? Q15: Why is my fatigue level so high? Q16: I keep waking up in the middle of the night, and I’ve tried the yoga methods you suggested, but they don’t seem to work?
5	Q17: I need to get up early tomorrow. How should I prepare tonight? Q18: Can melatonin help improve my sleep? Q19: I had coffee last night, and it greatly affected my sleep, but your measured data doesn’t seem accurate. I actually slept around 9 h, not 8 h? Q20: What is heart rate variability (HRV), and why do you provide so much information about these parameters?

Subsequently, we evaluated the model’s responses to these real-world questions using Claude Sonnet 3.7 and human experts. The evaluations focused on three key aspects: personalization, relevance, and completeness. The human expert scores for these dimensions were 4.3, 4.5, and 4.3, respectively, while the LLM scores were 4.4, 4.4, and 4.5. The results are summarized in Table 15. The evaluation results of Claude Sonnet 3.7 are highly consistent with those of human experts in all dimensions.

**TABLE 15:** Evaluation scores for model responses to real-world questions.

Evaluator	Pers.	Rel.	Comp.
Human expert	4.3	4.5	4.3
Claude sonnet 3.7	4.4	4.4	4.5

## 5 Discussion

### 5.1 Advantages over existing methods

The proposed framework demonstrates clear advantages over existing approaches in terms of both technical performance and practical deployment. In data synthesis, our method achieves a KL divergence of 0.85, indicating high fidelity to the original data distribution—an essential property for generating clinically meaningful HRV parameters. While the HSIC value (0.37) is lower than that of Gaussian Copula (0.60) and SMOTE (0.52), this reflects a deliberate emphasis on distributional accuracy over diversity, which is critical for reliable sleep report generation. In contrast, many existing methods prioritize diversity at the expense of clinical faithfulness. Furthermore, the personalized questions generated by our framework received high average scores for personalization (4.2), relevance (4.1), and diversity (4.1), outperforming traditional data augmentation techniques and demonstrating strong suitability for downstream analytical and interactive tasks.

For personalized inference, our model was validated across diverse populations and sleep scenarios, consistently matching or exceeding the performance of the teacher model (Qwen-max) and outperforming baseline models. For example, on the DREAMT dataset, our model achieved higher personalization (4.6) and relevance (4.7) scores compared to both the fine-tuned 0.5 billion-parameter and 1.5 billion-parameter models. The MoE LoRA architecture enables dynamic resource allocation, as shown by task-specific adapter activation patterns (Figure 6), ensuring robust and adaptive performance across heterogeneous datasets. This adaptability distinguishes our approach from conventional fine-tuning methods, which often lack such flexibility.

The framework’s practical value is confirmed by its successful deployment on the RK3588 edge device. It achieves a real-time inference speed of 16.6 tokens per second while maintaining a compact memory footprint of only 0.7 GB, demonstrating its suitability for resource-constrained environments. Our lightweight 0.5 billion-parameter base model offers a compelling balance between computational efficiency and analytical performance, making it a scalable and effective solution for real-world personalized health analysis.

### 5.2 Limitations and future work

Despite these strengths, several limitations warrant consideration. The framework’s reliance on user profiles for personalized inference implies that its effectiveness is influenced by the quality and completeness of input profiles. Enhancing robustness against incomplete or noisy profile data constitutes an important direction for future work. Additionally, while the current framework addresses multiple tasks effectively, its scalability to more complex or larger-scale applications remains to be fully validated. Whether the 0.5 billion-parameter base model, even with an increased number of adapters, advanced loss functions, or improved routing algorithms, can maintain high performance in more demanding scenarios is an open question. Future research will focus on hybrid data synthesis, advanced optimization strategies, and further architectural innovations to ensure continued scalability and adaptability.

## 6 Conclusion

This work presents a novel and efficient framework for personalized sleep analysis on edge devices, integrating profile-aided inference and adaptive data synthesis within a lightweight 0.5 billion-parameter model. The framework achieves a strong balance among accuracy, adaptability, and computational efficiency, enabling real-time, individualized health analysis in resource-constrained environments. Its modular design and successful deployment on edge hardware underscore its practical potential for wearable and mobile health applications.

By addressing key challenges in data scarcity, model efficiency, and user-specific inference, this study advances the state of the art in personalized health informatics. Looking ahead, the proposed approach lays a robust foundation for future innovations in scalable, secure, and accessible personalized healthcare, with the potential to expand beyond sleep analysis to broader domains of health monitoring and intervention.

## Data Availability

The datasets presented in this study can be found in online repositories. The names of the repository/repositories and accession number(s) can be found in the article/Supplementary Material.

## Footnotes

1
https://openai.com/index/whoop/

2
https://platform.openai.com/docs/guides/text?api-mode=responses

3
https://github.com/TUDB-Labs/MoE-PEFT/tree/main/moe_peft
