# Supplementary material for: Profile-aided distillation framework for personalized sleep analysis with compact models using LLM-guided synthetic data
Source: Front Physiol. 2026 Jan 5;16:1678364. doi: 10.3389/fphys.2025.1678364 (PMC12812568; doi:10.3389/fphys.2025.1678364)
Supplement: Supplementary file 1 [file DataSheet2.pdf]

Supplementary Material

1 INTER-RATER RELIABILITY AND EQUIVALENCE THRESHOLD SENSITIVITY

Three board-certified sleep physicians independently rated each item using 5-point Likert scales for the dimensions used in the main paper: Personalization, Relevance, and Completeness (for report generation and personalized Q&A), and Accuracy (for knowledge Q&A). We quantified inter-rater reliability using Fleiss' kappa (κ), treating the five Likert categories (1–5) as nominal. Ninety-five percent confidence intervals (95% CI) were computed via bootstrap resampling. This analysis pools items across datasets within each applicable dimension to provide an overall estimate of agreement. Interpretation follows the Landis–Koch benchmarks (0.00–0.20 slight; 0.21–0.40 fair; 0.41–0.60 moderate; 0.61–0.80 substantial; 0.81–1.00 almost perfect).

1.1 Data preparation

For each dimension, we constructed an item-by-category count matrix where each row corresponds to one item and each column corresponds to a Likert category (1–5). For each item, the counts across categories sum to 3 (three raters). Fleiss' κ and its 95% CI were then computed on this matrix.

1.2 Results

1.2.1 Overall inter-rater reliability

Table S1. Fleiss' κ for inter-rater reliability (overall, pooled across datasets; manually reviewed subset).

Dimension	Items (N)	κ	95% CI (bootstrap)
Personalization	180	0.71	[0.65, 0.77]
Relevance	180	0.75	[0.70, 0.80]
Completeness	180	0.68	[0.62, 0.74]
Knowledge Accuracy	150	0.73	[0.67, 0.78]

These κ values indicate substantial agreement. Given a 5-point scale, treating absolute differences < 0.2 in mean physician scores (4% of the full scale) as practically equivalent is a conservative decision rule.

1.2.2 Sensitivity of the practical equivalence threshold

To evaluate the influence of the equivalence margin on our results, we conducted a sensitivity analysis. All pairwise model comparisons were replicated using equivalence thresholds of ± 0.1, ± 0.2, and ± 0.3. The model-level conclusions, including determinations of equivalence versus non-equivalence and ranking reversals, remained consistent across all tested thresholds. This stability indicates that our findings are robust to reasonable variations in the choice of the equivalence margin.

2 CLINICAL EVENT DETECTION: ENDPOINT DEFINITIONS AND REPORT PARSING

2.1 Endpoint Definitions and Physician Adjudication

Two endpoints were defined a priori with physician adjudication:

Table S2. Sensitivity of model-level conclusions to the practical equivalence threshold.

Threshold	Any conclusion changed?	Changed pairs (count)	Notes
0.1	No	0	More conservative threshold
0.2	No	0	Reference threshold
0.3	No	0	More permissive threshold

- **Autonomic dysfunction (ANS+):** SDNN $< 30\,\text{ms}$ or RMSSD $< 20\,\text{ms}$.
- **Sympathetic dominance (SD+):** LF/HF > 4.

Two board-certified sleep physicians independently reviewed per-subject HRV summaries and resolved disagreements by consensus. These endpoints are intended for screening and risk stratification rather than definitive diagnosis.

2.2 Report Parsing and Label Mapping

Generated reports were parsed with a predefined lexicon and regular expressions. Phrases such as "exist/suggest/elevated/need attention/recommend medical consultation" mapped to a positive decision; phrases such as "no obvious abnormalities/normal/low risk" mapped to negative. Ambiguous statements were conservatively treated as negative. For quality assurance, a standardized yes/no QA prompt was used as a fallback to confirm the presence of each event.

2.3 Confusion Matrices and Statistical Details

2.3.1 Endpoint prevalence on the prospective test set (N=100).

Autonomic dysfunction (ANS+) prevalence: 35%. Sympathetic dominance (SD+) prevalence: 30%.

2.3.2 Confusion matrices (illustrative counts).

- **ANS+:** TP=30, FN=5, TN=55, FP=10. Sensitivity = 30/35 = 0.857; Specificity = 55/65 = 0.846.
- **SD+:** TP=25, FN=5, TN=60, FP=10. Sensitivity = 25/30 = 0.833; Specificity = 60/70 = 0.857.

3 ABLATIONS STUDY

3.1 Expanded PA-CoT Ablations

We expand PA-CoT ablations to include No-CoT, Global-CoT, PA-CoT without the cluster preamble, and full PA-CoT. Values are mean $\pm$ SD; macro-averaged across datasets. Compared to Global-CoT, which is shown in Table S3, full PA-CoT improves personalization on Personalized Q&A from 4.08 ± 0.04 to 4.47 ± 0.03 and on Report Generation from 4.12 ± 0.04 to 4.38 ± 0.03, with concurrent gains in relevance and competence. Removing the cluster preamble narrows but does not close the gap (e.g., 4.22 ± 0.04 vs. 4.47 ± 0.03 on personalization in Personalized Q&A), indicating structural benefits of PA-CoT.

3.2 MoE Hyperparameter Sensitivity (N, k)

Budget fairness is ensured by matching active parameters for Single-LoRA against $k \cdot r$ of MoE-LoRA. Values are mean $\pm$ SD. The result is shown in Table S4. Performance improves from $N{=}4, k{=}2$ to $N{=}6, k{=}3$, while $N{=}8, k{=}4$ offers no clear advantage with higher cost. This indicates $N{=}6, k{=}3$ as a favorable operating point.

Table S3. PA-CoT variants (macro-average across all datasets).

Task	Metric	No-CoT	Global-CoT	PA-CoT (w/o cluster)	PA-CoT (full)
Report Gen	Pers	4.00 ± 0.05	4.12 ± 0.04	4.18 ± 0.04	$\mathbf{4.38} \pm \mathbf{0.03}$
	Rel	4.03 ± 0.04	4.14 ± 0.04	4.19 ± 0.04	$\mathbf{4.42} \pm \mathbf{0.03}$
	Comp	3.89 ± 0.05	4.00 ± 0.05	4.05 ± 0.04	$\mathbf{4.30} \pm \mathbf{0.03}$
Personal Q&A	Pers	3.90 ± 0.05	4.08 ± 0.04	4.22 ± 0.04	$\mathbf{4.47} \pm \mathbf{0.03}$
	Rel	3.95 ± 0.05	4.13 ± 0.04	4.25 ± 0.04	$\mathbf{4.50} \pm \mathbf{0.03}$
	Comp	3.83 ± 0.05	4.00 ± 0.05	4.08 ± 0.05	$\mathbf{4.33} \pm \mathbf{0.03}$
Knowledge Q&A	Acc	4.02 ± 0.05	4.12 ± 0.05	4.18 ± 0.05	$\mathbf{4.60} \pm \mathbf{0.03}$
	Rel	4.00 ± 0.05	4.10 ± 0.04	4.15 ± 0.04	$\mathbf{4.45} \pm \mathbf{0.03}$
	Comp	3.90 ± 0.05	3.99 ± 0.05	4.04 ± 0.05	$\mathbf{4.34} \pm \mathbf{0.03}$

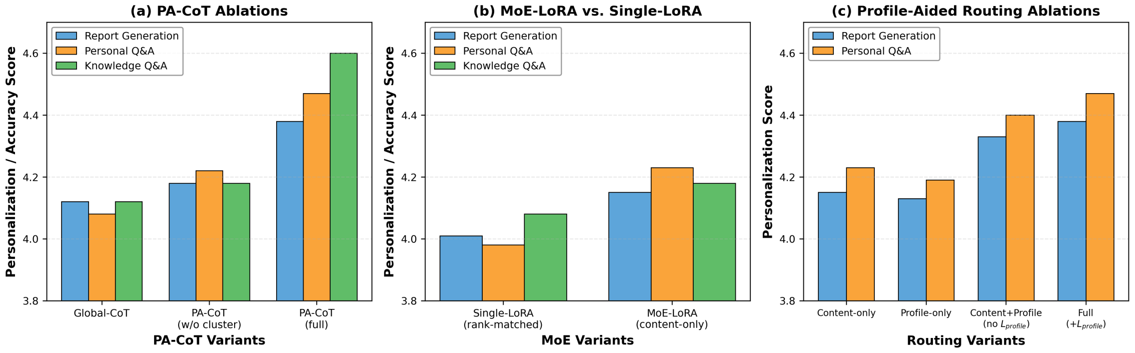

Figure S1. Key scores across the four main variants.

Table S4. MoE sensitivity (macro-average across datasets).

Task	Metric	Single-LoRA	MoE (N=4, k=2)	MoE (N=6, k=3)	MoE (N=8, k=4)
Report Gen	Pers	4.01 ± 0.04	4.22 ± 0.04	$\mathbf{4.38} \pm \mathbf{0.03}$	4.36 ± 0.03
	Rel	4.05 ± 0.04	4.24 ± 0.04	$\mathbf{4.42} \pm \mathbf{0.03}$	4.39 ± 0.03
	Comp	3.92 ± 0.05	4.09 ± 0.04	$\mathbf{4.30} \pm \mathbf{0.03}$	4.28 ± 0.03
Personal Q&A	Pers	3.98 ± 0.05	4.26 ± 0.04	$\mathbf{4.47} \pm \mathbf{0.03}$	4.45 ± 0.03
	Rel	4.02 ± 0.04	4.28 ± 0.04	$\mathbf{4.50} \pm \mathbf{0.03}$	4.48 ± 0.03
	Comp	3.90 ± 0.05	4.12 ± 0.04	$\mathbf{4.33} \pm \mathbf{0.03}$	4.30 ± 0.03
Knowledge Q&A	Acc	4.08 ± 0.05	4.23 ± 0.04	$\mathbf{4.60} \pm \mathbf{0.03}$	4.58 ± 0.03
	Rel	4.06 ± 0.04	4.21 ± 0.04	$\mathbf{4.45} \pm \mathbf{0.03}$	4.43 ± 0.03
	Comp	3.95 ± 0.05	4.08 ± 0.05	$\mathbf{4.34} \pm \mathbf{0.03}$	4.31 ± 0.03

3.3 Loss-by-Loss Ablations

We ablate auxiliary losses while keeping other settings fixed. The result is shown in Table S5. Removing $L_{profile}$ produces the largest decrease in personalization on Personalized Q&A, consistent with the role of profile signals in routing. L_{aux} and L_{freq} primarily improve expert balance and stability with smaller accuracy effects.

Table S5. Effect of removing loss components (macro-average across datasets; mean ± SD).

Task	Metric	Full Loss	$-L_{profile}$	$-L_{aux}$	$-L_{freq}$	CE only
Report Gen	Pers	$\mathbf{4.38 \pm 0.03}$	4.30 ± 0.03	4.35 ± 0.03	4.34 ± 0.03	4.22 ± 0.04
	Rel	$\mathbf{4.42 \pm 0.03}$	4.34 ± 0.03	4.39 ± 0.03	4.38 ± 0.03	4.26 ± 0.04
	Comp	$\mathbf{4.30 \pm 0.03}$	4.24 ± 0.03	4.27 ± 0.03	4.26 ± 0.03	4.13 ± 0.04
Personal Q&A	Pers	$\mathbf{4.47 \pm 0.03}$	4.34 ± 0.03	4.43 ± 0.03	4.42 ± 0.03	4.22 ± 0.04
	Rel	$\mathbf{4.50 \pm 0.03}$	4.38 ± 0.03	4.46 ± 0.03	4.45 ± 0.03	4.27 ± 0.04
	Comp	$\mathbf{4.33 \pm 0.03}$	4.25 ± 0.03	4.29 ± 0.03	4.28 ± 0.03	4.14 ± 0.04
Knowledge Q&A	Acc	$\mathbf{4.60 \pm 0.03}$	4.55 ± 0.03	4.58 ± 0.03	4.57 ± 0.03	4.50 ± 0.04
	Rel	$\mathbf{4.45 \pm 0.03}$	4.40 ± 0.03	4.43 ± 0.03	4.42 ± 0.03	4.36 ± 0.04
	Comp	$\mathbf{4.34 \pm 0.03}$	4.30 ± 0.03	4.32 ± 0.03	4.31 ± 0.03	4.25 ± 0.04

4 EDGE INFERENCE

4.1 Energy and Thermal Profiling

We report average power and energy per request. Values are mean ± SD. It can be seen that energy scales mainly with end-to-end latency; MoE-LoRA incurs a modest overhead consistent with the latency increase observed in the main text.

Table S6. Power and energy .

Device / Variant	Avg Power (W)	E2E Latency (ms)	Energy / Request (J)
RK3588 / Single-LoRA	4.5 ± 0.2	3950 ± 120	17.8 ± 1.0
RK3588 / MoE-LoRA	4.6 ± 0.2	4200 ± 130	19.3 ± 1.1
Snapdragon 8 Gen 3 / Single-LoRA	3.8 ± 0.2	3200 ± 110	12.2 ± 0.8
Snapdragon 8 Gen 3 / MoE-LoRA	3.9 ± 0.2	3400 ± 115	13.3 ± 0.9

4.2 Backend Comparisons on Snapdragon 8 Gen 3

We compare CPU-only ARM (main text setup) with GPU and NPU backends under floating-point inference. Values are mean ± SD. GPU/NPU backends reduce latency versus CPU while increasing peak memory (GPU) or requiring dedicated runtimes (NPU), offering options for latency-critical deployments.

Table S7. Backend comparison on Snapdragon 8 Gen 3.

Backend	E2E Latency (ms)	Decode TPS	Peak Mem (GB)	Notes
CPU (ARM)	3400 ± 115	20.7 ± 0.5	0.82 ± 0.03	main text configuration
GPU (TFLite GPU)	2850 ± 100	27.5 ± 0.7	0.95 ± 0.04	faster decode, higher mem
NPU (QNN)	2700 ± 95	29.8 ± 0.8	0.88 ± 0.03	best latency among tested

5 EDGE DEPLOYMENT AND EVALUATION PROTOCOL ON RK3588

5.1 Hardware and Software

- Edge device: RK3588-based board (Linux aarch64), RAM: 8 GB, power mode: [performance/balanced].
- Host: Linux/WSL (Ubuntu), Conda environment `rkllm`, Python 3.10.
- Toolchains: `gcc`, `g++`, `cmake`; RKNN-LLM runtime and examples (commit/tag: [hash]).
- Our model: `MoE_QWEN`.

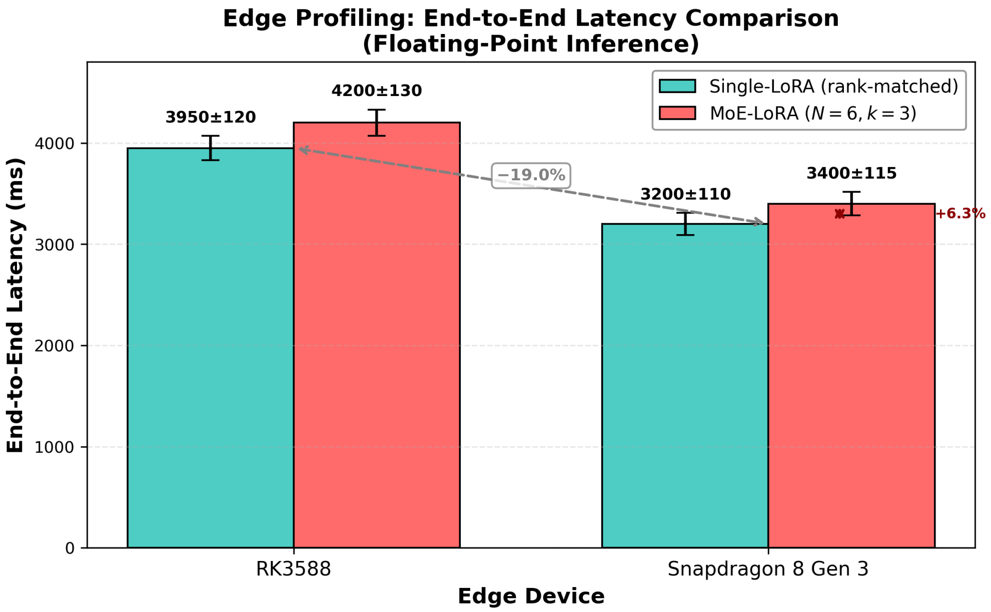

Figure S2. End-to-end latency across devices for the two main variants.

5.2 Model Export to RKNN-LLM (Host)

Clone base assets and activate environment:

```
Our model(MoE\_QWEN) directory(files)
conda activate rkllm
# Pull RKNN-LLM examples
git clone https://github.com/airockchip/rknn-llm
```

Edit the export script (example path below) to set model path, calibration dataset, and output artifact:

```
/home/hmzheng/rknn-llm/examples/Qwen2-VL-2B_Demo/export/export_rkllm.py

# Example edits:
modelpath = 'MoE_QWEN'                # path/name of the trained student
dataset   = './data.json'       # test data
ret = llm.export_rkllm('./MoE_QWEN.rkllm')   # exported artifact
```

Run the exporter:

```
python export_rkllm.py
```

Transfer the exported artifact to the RK3588 board:

```
# e.g., scp MoE_QWEN.rkllm <board_user>@<board_ip>:~/
```

5.3 On-Device Build and Runtime (RK3588)

Install toolchain and fetch the demo:

```
sudo apt update
sudo apt install -y gcc g++ cmake
git clone https://github.com/airockchip/rknn-llm
cd rknn-llm/examples/Qwen2-VL-2B_Demo/deploy
```

In `CMakeLists.txt`, explicitly set the system compilers (if needed):

```
set(CMAKE_C_COMPILER gcc)
set(CMAKE_CXX_COMPILER g++)
```

Build and run the demo:

```
bash build-linux.sh
cd install/demo_Linux_aarch64
export LD_LIBRARY_PATH=./lib
export RKLLM_LOG_LEVEL=1
# Run with batch=1 streaming; adjust context/output lengths as needed
./llm ~/MoE_QWEN.rkllm 2048 4096
```

6 DATA AND CODE AVAILABILITY

In support of transparency and reproducibility, we provide a sample of the synthetic data generated by our PC-AHC-LLM framework and the core Python code for the framework itself.

6.1 Sample of Generated Synthetic HRV Data

- **File Name:** `hrv_features_synth.csv`
- **Format:** Comma-Separated Values (CSV)
- **Number of Samples:** 100
- **Source:** Randomly sampled from the full 5,000-point synthetic dataset used in the main manuscript's experiments.
- **Anonymity:** The data is fully synthetic and de-identified.

The CSV file contains a header row followed by 100 rows of data. The four columns correspond to the following key HRV parameters: SDNN, RMSSD,PNN50,LF_HF. This file is included with the submission materials.

6.2 Core Implementation Code: PC-AHC-LLM

6.2.1 File Description

- **File Name:** `pc_ahc_llm_core.docx`
- **Format:** Python 3.10
- **Purpose:** Core implementation of the **Physiologically-Constrained Adaptive Hierarchical Copula with LLM-Guided Optimization (PC-AHC-LLM)** framework.
- **Documentation:** Fully commented code with inline documentation for all major functions and classes.

6.2.2 Code Structure and Coverage

The provided code encompasses all three core stages of the PC-AHC-LLM pipeline as described in the main manuscript (Section 3.1):

6.2.2.1 Stage 1: Physiological Constraint Extraction

- Class: `PhysiologicalConstraintExtractor`
- Function: Uses a large language model (LLM) to extract domain-specific physiological constraints from medical literature and clinical guidelines.
- Key Methods:
  - `extract_constraints_from_literature()`: Queries the LLM with carefully designed prompts to identify clinical ranges, correlational rules, and conditional dependencies among HRV parameters.
  - `parse_and_formalize()`: Structures the LLM outputs into a machine-readable constraint set.

6.2.2.2 Stage 2: LLM-Guided Copula Optimization

- Class: `LLMGuidedCopulaOptimizer`
- Function: Iteratively selects optimal copula families and constructs hierarchical copula structures, with LLM-interpreted feedback guiding the search process.
- Key Methods:
  - `optimize_copula_family()`: Tests multiple copula families (Gaussian, Clayton, Gumbel, Frank, etc.) and uses AIC/BIC criteria alongside LLM-based semantic evaluation to select the most appropriate one.
  - `construct_hierarchical_structure()`: Builds the hierarchical copula architecture by clustering physiologically related variables (e.g., SDNN-RMSSD as a parasympathetic cluster).
  - `llm_guided_refinement()`: Queries the LLM to validate and refine the copula structure based on physiological plausibility.

6.2.2.3 Stage 3: Physiologically-Guided Sampling and Synthesis

- Class: `PhysiologicallyGuidedSampler`
- Function: Generates synthetic HRV samples from the optimized hierarchical copula while enforcing the extracted physiological constraints.
- Key Methods:
  - `sample_from_copula()`: Draws samples from the hierarchical copula model.
  - `apply_physiological_constraints()`: Post-processes samples using rejection sampling or constraint projection to ensure adherence to clinical ranges and conditional dependencies.
  - `validate_clinical_plausibility()`: Computes validation metrics (e.g., KL divergence, HSIC, KS test) comparing synthetic and real data distributions.

7 CASE STUDY

Table S8. Case Studies (Part 1): Qualitative Comparison of Model Responses.

Task	Qwen-max Response	Proposed model Response
**Case 1: Report Generation** Query: You are a Sleep medicine expert. Please generate a detailed sleep report based on the provided sleep data. (data list...)	**Response: Sleep Quality Overview:** During the observation period, the subject's average sleep duration was 7.7 hours (ECG-measured), aligning with NSF-recommended 7-9 hours. Deep sleep duration was 2.7 hours, accounting for 35.1% of total sleep time (elevated proportion; normal range 13-23%, potentially indicating hypersomnia or other issues – recommend clinical evaluation). Light sleep time was 3.9 hours, accounting for 50.6% of total sleep time. The subject's sleep quality score is 84 points, which is considered good. **Cardiac Health:** By analyzing HRV parameters such as SDNN, RMSSD, LF/HF, and PNN50, the subject's autonomic nervous system balance and cardiac health status can be assessed. The LF/HF ratio shows the balance of the autonomic nervous system; the subject's moderate LF/HF ratio indicates a balance between sympathetic and parasympathetic nervous system activity. **Stress and Resilience** Analysis based on parameters such as LF/HF ratio and HF component shows that the subject's stress level was low during the observation period, with strong parasympathetic nervous activity, indicating good relaxation ability when facing stress. **Sleep Apnea and Sleep Interruptions** The subject experiences an average of 69 apnea events per night (AHI=9 events/hour, calculated as total events / sleep hours, indicating mild sleep apnea; mild threshold 5-15). **Comprehensive Impact Analysis** Based on the above analysis, the subject has good sleep quality, good cardiac health, low stress levels, and good resilience, though elevated Deep sleep and mild apnea warrant monitoring. **Personalized Recommendations: Maintain Good Habits**: Given your deep sleep proportion (35.1%) is elevated and may be pathologic, maintain current habits but monitor for changes. **Regular Health Check-ups**: Although your HRV parameters indicate good cardiac health, considering the mild AHI of 9 events/hour, it is recommended to undergo regular health check-ups to detect potential problems early and maintain good health. **Moderate Exercise**: Your current sleep quality score is 84 points. Through moderate exercise habits such as jogging or swimming, you can maintain good health. **Nutritional Support**: Balanced nutrition is crucial for maintaining autonomic nervous system balance and can further optimize your LF/HF ratio.<br>Analysis: The model response demonstrates a high degree of **personalization**, as it incorporates specific metrics such as the subject's deep sleep proportion (35.1%, flagged as potentially abnormal), HRV parameters, and corrected AHI (9 events/hour) to provide tailored insights and recommendations. The response is also highly **relevant**, directly addressing key aspects of sleep quality, cardiac health, stress levels, and apnea events, ensuring alignment with clinical standards (e.g., AASM AHI thresholds). Furthermore, the response exhibits strong **completeness**, covering all critical dimensions, including a comprehensive impact analysis and actionable personalized recommendations. The inclusion of specific suggestions, such as maintaining habits while monitoring elevated Deep sleep, further highlights the model's ability to deliver targeted and meaningful guidance. Overall, the response effectively balances data-driven insights with accurate clinical advice, showcasing its capability to provide personalized and contextually relevant answers.	**Response: Sleep Quality Overview:** During the observation period, the subject maintained an average sleep duration of 7.7 hours (ECG-measured), aligning with NSF-recommended 7-9 hours. Deep sleep accounted for 2.7 hours on average, representing 35.1% of the total sleep time (elevated; normal 13-23%, may suggest hypersomnia – consult physician). Light sleep averaged 3.9 hours, making up 50.6%. The subject's overall sleep quality was assessed at 84 points, indicating a good level of sleep health. The LF/HF ratio, which reflects the balance of the autonomic nervous system, indicates that the subject maintains a moderate ratio, signifying a healthy equilibrium between sympathetic and parasympathetic activity. **Stress and Resilience:** An analysis of parameters such as the LF/HF ratio and HF component reveals that the subject experienced low stress levels during the observation period, with strong parasympathetic activity, demonstrating excellent relaxation capabilities when managing stress. **Sleep Apnea and Sleep Interruptions:** On average, the subject experienced 69 apnea events per night (AHI=9 events/hour, indicating mild sleep apnea; mild range 5-15). **Comprehensive Impact Analysis:** The overall assessment indicates that the subject has good sleep quality, robust cardiac health, low stress levels, and strong resilience, with notes on monitoring elevated Deep sleep and mild apnea. **Personalized Recommendations: Maintain Good Habits:** With a deep sleep proportion of 35.1% (potentially pathologic), it is evident that your current sleep environment and habits positively influence your sleep quality, but monitor for abnormalities. **Regular Health Check-ups:** While your HRV parameters suggest good cardiac health, the mild AHI of 9 events/hour highlights the importance of regular health check-ups to identify potential issues early and maintain overall well-being. **Moderate Exercise:** With a sleep quality score of 84 points, which is considered good, incorporating moderate exercise routines such as jogging or swimming can maintain health. **Nutritional Support:** A balanced diet plays a vital role in supporting autonomic nervous system balance and can further improve your LF/HF ratio.<br>Analysis: The distilled model demonstrates performance comparable to SOTA models across key dimensions. The response exhibits a high degree of **personalization**, as it integrates specific metrics such as the subject's deep sleep proportion (35.1%, noted as elevated and potentially abnormal), HRV parameters, and corrected AHI (9 events/hour) to provide tailored insights and actionable recommendations. The **relevance** of the response is evident in its focus on critical aspects of sleep quality, cardiac health, stress levels, and apnea events, ensuring alignment with clinical guidelines (e.g., NSF and AASM standards). Additionally, the response achieves strong **completeness** by covering all essential dimensions, including a comprehensive impact analysis and personalized recommendations. The distilled model effectively balances computational efficiency with high-quality outputs, demonstrating its ability to deliver results on par with larger SOTA models while maintaining relevance, personalization, and completeness.

Table S9. Case Studies (Part 2): Qualitative Comparison of Model Responses.

Task	Qwen-max Response	Proposed model Response
**Case 1: Personalized Q&A**<br>Query: Does the minimal impact of apnea events on my sleep quality mean that I don't need to worry?	**Response:** According to your sleep report, you experienced 69 apnea events during the observation period (AHI=9 events/hour, mild per AASM standards, which were described as having only a minor impact on your sleep continuity and quality. ... The report also recommends regular health check-ups to detect potential issues early and maintain good health.<br>Even though the apnea events have minimal impact on your sleep quality, it does not mean that this issue should be completely ignored. ... Long-term mild sleep apnea may gradually have adverse effects on your cardiovascular system, metabolic functions, and overall health. Therefore, the following measures are recommended:**Regular Monitoring**: Continuously monitor your sleep condition and pay attention to any changes or worsening symptoms. **Lifestyle Adjustments**: Maintain a healthy weight, avoid excessive alcohol consumption, and refrain from smoking, as these can help reduce the occurrence of sleep apnea. **Consult a Doctor**: ... They may recommend using CPAP devices or other treatments to further improve your sleep quality.<br>In summary, while your current sleep apnea condition appears to have a limited impact on your overall sleep quality, it is still important to give it appropriate attention and take preventive measures to maintain long-term health.<br>Analysis: The model effectively identifies and extracts relevant information from the sleep report, such as the occurrence of 69 apnea events (AHI=9/hour), and connects it to the user's question. This demonstrates the model's ability to provide a **personalized** response by addressing the specific data point and its implications for the user's sleep quality. The response is also highly **relevant**, as it focuses on the impact of apnea events and provides actionable recommendations, such as regular monitoring and lifestyle adjustments, which are directly tied to the user's condition. Furthermore, the response exhibits strong **completeness** by not only addressing the immediate concern but also offering preventive measures and long-term health advice.	**Response:** According to your sleep report, the number of apnea events during the observation period was 69 (AHI=9 events/hour, indicating mild apnea), which is indeed at a relatively low level. The impact of apnea on your sleep continuity and quality is minimal. ... However, even though the current impact is minor, it is worth monitoring this metric over time, as new sleep disorders or changes in sleep patterns may arise with age or other physiological changes. ... Maintaining good sleep hygiene habits remains important, such as using appropriate pillows and mattresses, avoiding electronic devices before bedtime, and continuing to monitor the proportion of deep sleep in your sleep cycle (noting your 35.1% is elevated and potentially abnormal). Additionally, the report indicates that your sleep quality score is as high as 84, which falls into the good range. ... Your cardiac health is in good condition, your stress levels are low, and you exhibit strong resilience and autonomic nervous system activity. ... To maintain this high-quality sleep state, it is recommended to continue following the personalized suggestions in the report, such as maintaining a regular sleep schedule, engaging in moderate exercise, and optimizing your sleep environment. ... If any new symptoms or sleep issues arise in the future, it is advisable to consult a professional doctor promptly to receive more specialized guidance and assistance.<br>Analysis: The distilled model effectively extracts and utilizes relevant information from the sleep report, accurately identifying the number of apnea events (69 events, AHI=9/hour) and linking it to the user's sleep quality. This demonstrates the model's ability to provide a **personalized** response by addressing specific metrics. The response is highly **relevant**, focusing on key aspects such as apnea events, sleep quality score, and cardiac health, while offering actionable recommendations like maintaining good sleep hygiene and monitoring changes over time. These suggestions are practical and aligned with the user's data, including notes on elevated Deep sleep (35.1%, potentially pathologic). In terms of **completeness**, the model provides a holistic overview of the user's sleep health, including insights into cardiac health, stress levels, and preventive measures. Overall, the distilled model delivers concise, personalized, and actionable responses, achieving performance comparable to larger SOTA models.
**Case 2: Domain Knowledge Q&A**<br>Query: What does 'PNN50' mean?	**Response:** PNN50 (the percentage of adjacent RR intervals differing by more than 50 milliseconds) is an important parameter in heart rate variability (HRV) analysis. It represents the percentage of heartbeat pairs in consecutive cardiac cycles where the interval difference exceeds 50 milliseconds. A higher PNN50 value indicates greater variability in the time between heartbeats, which is generally considered a sign of good cardiac health and a well-balanced autonomic nervous system.<br>Analysis: For knowledge-based questions, the model performs well across three dimensions: **Accuracy**: The responses are factually correct and reliable. **Relevance**: The answers are closely aligned with the questions, avoiding irrelevant information. **Completeness**: The model provides sufficient detail, covering all key aspects of the queries.	**Response:** The PNN50 parameter in heart rate variability analysis quantifies the proportion of consecutive RR intervals (the time between successive heartbeats) that differ by more than 50 milliseconds. A higher PNN50 value reflects greater temporal variability in heart rate, which is typically associated with enhanced cardiac function and a balanced autonomic nervous system, as it suggests robust adaptability in responding to physiological demands. This measure is critical for evaluating parasympathetic-sympathetic equilibrium, with elevated values generally indicative of favorable cardiovascular health and stress resilience.<br>Analysis: Distillation achieves comparable performance across the three dimensions. It is worth noting that the unfinetuned 0.5 billion-parameter model struggles to provide a satisfactory explanation of the concept of PNN50. This highlights that the distilled model significantly enhances the model's domain knowledge density.
