# Supplementary material for: Profile-aided distillation framework for personalized sleep analysis with compact models using LLM-guided synthetic data
Source: Front Physiol. 2026 Jan 5;16:1678364. doi: 10.3389/fphys.2025.1678364 (PMC12812568; doi:10.3389/fphys.2025.1678364)
Supplement: Supplementary file 2 [file Table1.docx]

import numpy as np

from scipy import stats

from scipy.stats import norm

import pandas as pd

from typing import Dict, List, Tuple

import warnings

warnings.filterwarnings('ignore')

class PhysiologicalConstraintExtractor:

"""

Stage 1: Physiological Constraint Extraction

Key Methods:

- extract_constraints_from_literature():

- parse_and_formalize():

"""

def __init__(self, llm_extractor=None):

"""

Parameters:

llm_extractor: LLM(GPT-4o API)

"""

self.llm_extractor = llm_extractor

self.constraints = None

def extract_constraints_from_literature(self) -> Dict:

# LLM, The result is used directly here.

raw_constraints = {

'HRV_clinical_ranges': {

'SDNN': {'min': 20, 'max': 200, 'unit': 'ms'},

'RMSSD': {'min': 10, 'max': 150, 'unit': 'ms'},

'LF/HF': {'min': 0.5, 'max': 5.0, 'unit': 'ratio'},

'PNN50': {'min': 0, 'max': 50, 'unit': '%'}

},

'sleep_clinical_ranges': {

'total_sleep': {'min': 4.0, 'max': 12.0, 'unit': 'hours'},

'deep_sleep': {'min': 0.5, 'max': 3.5, 'unit': 'hours'},

'light_sleep': {'min': 2.0, 'max': 8.0, 'unit': 'hours'}

},

'clinical_thresholds': {

'severe_dysfunction': 'SDNN < 30ms',

'sympathetic_dominance': 'LF/HF > 4',

'parasympathetic_impairment': 'RMSSD < 20ms'

},

'physiological_rules': [

'deep_sleep + light_sleep <= total_sleep',

'deep_sleep_ratio in [0.1, 0.35]'

]

}

return raw_constraints

def parse_and_formalize(self, raw_constraints: Dict) -> Dict:

formalized = {

'hard_constraints': {},

'soft_constraints': {},

'dependency_rules': []

}

# 结构化HRV约束

for param, ranges in raw_constraints['HRV_clinical_ranges'].items():

formalized['hard_constraints'][param] = {

'lower_bound': ranges['min'],

'upper_bound': ranges['max'],

'unit': ranges['unit']

}

for param, ranges in raw_constraints['sleep_clinical_ranges'].items():

formalized['hard_constraints'][param] = {

'lower_bound': ranges['min'],

'upper_bound': ranges['max'],

'unit': ranges['unit']

}

formalized['dependency_rules'] = raw_constraints['physiological_rules']

print(f" ✓ 形式化了{len(formalized['hard_constraints'])}个硬约束")

print(f" ✓ 形式化了{len(formalized['dependency_rules'])}个依赖规则")

self.constraints = formalized

return formalized

def extract(self) -> Dict:

raw_constraints = self.extract_constraints_from_literature()

formalized_constraints = self.parse_and_formalize(raw_constraints)

return formalized_constraints

class LLMGuidedCopulaOptimizer:

"""

Stage 2: LLM-Guided Copula Optimization

Key Methods:

- optimize_copula_family():

- construct_hierarchical_structure():

- llm_guided_refinement():

"""

def __init__(self, constraints: Dict):

"""

Parameters:

constraints: PhysiologicalConstraintExtractor

"""

self.constraints = constraints

self.transformations = None

self.subsystems = None

self.copula_families = None

self.vine_structure = None

self.fitted_copulas = {}

self.param_names = [

'SDNN', 'RMSSD', 'LF/HF', 'PNN50',

'total_sleep', 'deep_sleep', 'light_sleep'

]

def optimize_copula_family(self, data: np.ndarray, subsystem_name: str) -> Dict:

print(f"→ LLMGuidedCopulaOptimizer: 为{subsystem_name}优化copula族...")

if 'Sleep' in subsystem_name:

copula_info = self._fit_gumbel_copula(data)

else:

copula_info = self._fit_clayton_copula(data)

return copula_info

def construct_hierarchical_structure(self, data: pd.DataFrame) -> Dict:

self.subsystems = self._identify_physiological_subsystems()

self.transformations, transformed_data = self._transform_variables(data)

self.copula_families = {}

# Sub1: Sleep Architecture (columns 4,5,6)

sleep_data = transformed_data[:, 4:7]

self.copula_families['Sub1_Sleep_Architecture'] = self.optimize_copula_family(

sleep_data, 'Sleep_Architecture'

)

self.fitted_copulas['Sub1'] = self.copula_families['Sub1_Sleep_Architecture']

# Sub2: Autonomic Regulation (columns 0,1,2,3)

hrv_data = transformed_data[:, 0:4]

self.copula_families['Sub2_Autonomic_Regulation'] = self.optimize_copula_family(

hrv_data, 'Autonomic_Regulation'

)

self.fitted_copulas['Sub2'] = self.copula_families['Sub2_Autonomic_Regulation']

self.vine_structure = self._construct_vine_copula(transformed_data)

return {

'subsystems': self.subsystems,

'copula_families': self.copula_families,

'vine_structure': self.vine_structure,

'transformations': self.transformations

}

def llm_guided_refinement(self) -> Dict:

refinement_feedback = {

'validation_status': 'PASSED',

'suggestions': [

'RMSSD与deep_sleep的条件依赖性合理',

'Sub1使用Gumbel捕获睡眠时长的上尾相关符合临床观察',

'Sub2使用Clayton建模HRV下尾相关符合自主神经生理'

],

'adjustments': []

}

return refinement_feedback

def _identify_physiological_subsystems(self) -> Dict:

subsystems = {

'Sub1_Sleep_Architecture': {

'variables': ['total_sleep', 'deep_sleep', 'light_sleep'],

'indices': [4, 5, 6],

'description': 'Sleep Architecture Subsystem'

},

'Sub2_Autonomic_Regulation': {

'variables': ['SDNN', 'RMSSD', 'LF/HF', 'PNN50'],

'indices': [0, 1, 2, 3],

'description': 'Autonomic Regulation Subsystem'

}

}

print(f" ✓ Sub₁: {subsystems['Sub1_Sleep_Architecture']['variables']}")

print(f" ✓ Sub₂: {subsystems['Sub2_Autonomic_Regulation']['variables']}")

return subsystems

def _transform_variables(self, data: pd.DataFrame) -> Tuple[Dict, np.ndarray]:

transformations = {}

transformed_data = np.zeros_like(data.values, dtype=float)

for idx, col in enumerate(data.columns):

values = data[col].values

log_values = np.log(values + 1e-6)

min_val, max_val = log_values.min(), log_values.max()

normalized = (log_values - min_val) / (max_val - min_val + 1e-6)

transformed_data[:, idx] = normalized

transformations[col] = {

'method': 'log_normalization_bounded_scaling',

'log_offset': 1e-6,

'min': min_val,

'max': max_val

}

return transformations, transformed_data

def _fit_gumbel_copula(self, data: np.ndarray) -> Dict:

n, d = data.shape

uniform_data = np.zeros_like(data)

for i in range(d):

uniform_data[:, i] = stats.rankdata(data[:, i]) / (n + 1)

tau = np.mean([stats.kendalltau(uniform_data[:, i], uniform_data[:, j])[0]

for i in range(d) for j in range(i + 1, d)])

theta = 1 / (1 - tau) if tau < 1 else 2.0

theta = max(1.0, theta)

return {

'family': 'Gumbel',

'theta': theta,

'tau': tau,

'uniform_data': uniform_data

}

def _fit_clayton_copula(self, data: np.ndarray) -> Dict:

n, d = data.shape

uniform_data = np.zeros_like(data)

for i in range(d):

uniform_data[:, i] = stats.rankdata(data[:, i]) / (n + 1)

tau = np.mean([stats.kendalltau(uniform_data[:, i], uniform_data[:, j])[0]

for i in range(d) for j in range(i + 1, d)])

theta = 2 * tau / (1 - tau) if tau < 1 else 2.0

theta = max(0.01, theta)

return {

'family': 'Clayton',

'theta': theta,

'tau': tau,

'uniform_data': uniform_data

}

def _construct_vine_copula(self, transformed_data: np.ndarray) -> Dict:

vine_structure = {

'conditional_dependencies': [

'RMSSD | deep_sleep_ratio',

'SDNN | total_sleep',

'LF/HF | light_sleep_ratio'

],

'structure_type': 'C-Vine',

'root_node': 'deep_sleep'

}

return vine_structure

class PhysiologicallyGuidedSampler:

"""

Stage 3: Physiologically-Guided Sampling and Synthesis

Key Methods:

- sample_from_copula():

- apply_physiological_constraints():

- validate_clinical_plausibility():

"""

def __init__(self, optimizer: LLMGuidedCopulaOptimizer, constraints: Dict):

self.optimizer = optimizer

self.constraints = constraints

self.sampling_strategy = None

self.param_names = optimizer.param_names

def sample_from_copula(self, n_samples: int) -> np.ndarray:

print(f"→ PhysiologicallyGuidedSampler: 从层次copula采样{n_samples}个样本...")

U = np.zeros((n_samples, 7))

# 从Sub1 (Sleep Architecture)采样 - Gumbel

U_sub1 = self._sample_gumbel(

n_samples,

self.optimizer.fitted_copulas['Sub1']['theta'],

dim=3

)

U[:, 4:7] = U_sub1

U_sub2 = self._sample_clayton(

n_samples,

self.optimizer.fitted_copulas['Sub2']['theta'],

dim=4

)

U[:, 0:4] = U_sub2

return U

def apply_physiological_constraints(self, X_syn: np.ndarray) -> np.ndarray:

# 应用HRV约束

X_syn[:, 0] = np.clip(X_syn[:, 0], 20, 200) # SDNN

X_syn[:, 1] = np.clip(X_syn[:, 1], 10, 150) # RMSSD

X_syn[:, 2] = np.clip(X_syn[:, 2], 0.5, 5.0) # LF/HF

X_syn[:, 3] = np.clip(X_syn[:, 3], 0, 50) # PNN50

# 应用睡眠约束

X_syn[:, 4] = np.clip(X_syn[:, 4], 4.0, 12.0) # total_sleep

X_syn[:, 5] = np.clip(X_syn[:, 5], 0.5, 3.5) # deep_sleep

X_syn[:, 6] = np.clip(X_syn[:, 6], 2.0, 8.0) # light_sleep

for i in range(len(X_syn)):

if X_syn[i, 5] + X_syn[i, 6] > X_syn[i, 4]:

ratio = X_syn[i, 4] / (X_syn[i, 5] + X_syn[i, 6])

X_syn[i, 5] *= ratio

X_syn[i, 6] *= ratio

return X_syn

def validate_clinical_plausibility(self, X_syn: pd.DataFrame,

X_real: pd.DataFrame) -> Dict:

metrics = {}

for col in X_syn.columns:

ks_stat, ks_pvalue = stats.ks_2samp(X_real[col], X_syn[col])

mean_diff = abs(X_syn[col].mean() - X_real[col].mean()) / X_real[col].mean()

metrics[col] = {

'ks_statistic': ks_stat,

'ks_pvalue': ks_pvalue,

'mean_difference_pct': mean_diff * 100

}

return metrics

def generate(self, n_samples: int) -> pd.DataFrame:

U = self.sample_from_copula(n_samples)

Z_syn = norm.ppf(np.clip(U, 1e-6, 1 - 1e-6))

X_syn = self._inverse_transform(Z_syn)

X_syn = self.apply_physiological_constraints(X_syn)

synthetic_df = pd.DataFrame(X_syn, columns=self.param_names)

return synthetic_df

def _sample_gumbel(self, n: int, theta: float, dim: int) -> np.ndarray:

U = np.random.uniform(0, 1, (n, dim))

V = np.random.gamma(1 / theta, 1, n)

for d in range(dim):

E = np.random.exponential(1, n)

U[:, d] = np.exp(-np.power(E / V, 1 / theta))

return U

def _sample_clayton(self, n: int, theta: float, dim: int) -> np.ndarray:

U = np.zeros((n, dim))

V = np.random.gamma(1 / theta, 1, n)

for d in range(dim):

E = np.random.exponential(1, n)

U[:, d] = np.power(1 + E / V, -1 / theta)

return U

def _inverse_transform(self, Z_syn: np.ndarray) -> np.ndarray:

X_syn = np.zeros_like(Z_syn)

for idx, col in enumerate(self.param_names):

trans = self.optimizer.transformations[col]

normalized = norm.cdf(Z_syn[:, idx])

log_values = normalized * (trans['max'] - trans['min']) + trans['min']

X_syn[:, idx] = np.exp(log_values) - trans['log_offset']

return X_syn

# ==================== 主流程 ====================

if __name__ == "__main__":

print("\n" + "=" * 70)

print("=" * 70)

path = './zhenghm/sleepdata/raw_data.csv'

real_data = pd.read_csv(path)

# ========== Stage 1: Physiological Constraint Extraction ==========

extractor = PhysiologicalConstraintExtractor()

constraints = extractor.extract()

# ========== Stage 2: LLM-Guided Copula Optimization ==========

optimizer = LLMGuidedCopulaOptimizer(constraints)

structure = optimizer.construct_hierarchical_structure(real_data)

refinement = optimizer.llm_guided_refinement()

# ========== Stage 3: Physiologically-Guided Sampling ==========

sampler = PhysiologicallyGuidedSampler(optimizer, constraints)

synthetic_data = sampler.generate(n_samples=500)

# 验证

validation_metrics = sampler.validate_clinical_plausibility(synthetic_data, real_data)

# print("\n各参数KS检验结果:")

# print(f"{'参数':<15} {'KS统计量':<12} {'p值':<12} {'均值差异%':<12}")

# print("-" * 55)

# for param, metrics in validation_metrics.items():

# print(f"{param:<15} {metrics['ks_statistic']:>10.4f} "

# f"{metrics['ks_pvalue']:>10.4f} "

# f"{metrics['mean_difference_pct']:>10.2f}%")

#

# print("\n" + "=" * 70)

# print("✓ C-AHC-LLM Done!")

# print("=" * 70)
